# The Antitumor and Sorafenib-resistant Reversal Effects of Ursolic Acid on Hepatocellular Carcinoma via Targeting ING5

**DOI:** 10.7150/ijbs.97720

**Published:** 2024-08-05

**Authors:** Yin-Jie Fan, Fu-Zhi Pan, Zheng-Guo Cui, Hua-Chuan Zheng

**Affiliations:** 1 College of Integrated Chinese and Western Medicine, Liaoning University of Traditional Chinese Medicine, Shenyang 110001, Liaoning Province, China.; 2 Department of Ultrasound Medicine, Liaoning Cancer Hospital, Shenyang 110001, Liaoning Province, China.; 3 Department of Environmental Health, University of Fukui School of Medical Sciences, Fukui 910-1193, Japan.; 4 Cancer Center, The First Affiliated Hospital of Jinzhou Medical University, Jinzhou 121001, Liaoning Province, China.; 5 Department of Oncology and Central Laboratory, The Affiliated Hospital of Chengde Medical University, Chengde 067000, Hebei Province, China.

**Keywords:** Ursolic acid, Hepatocellular carcinoma, ING5, Sorafenib resistance, Spontaneous hepatocellular carcinoma model.

## Abstract

Inhibitor of growth 5 (ING5) has been reported to be involved in the malignant progression of cancers. Ursolic acid (UA) has shown remarkable antitumor effects. However, its antitumor mechanisms regarding of ING5 in hepatocellular carcinoma (HCC) remain unclear. Herein, we found that UA significantly suppressed the proliferation, anti-apoptosis, migration and invasion of HCC cells. In addition, ING5 expression in HCC cells treated with UA was obviously downregulated in a concentration- and time-dependent manner. Additionally, the pro-oncogenic role of ING5 was confirmed in HCC cells. Further investigation revealed that UA exerted antitumor effects on HCC by inhibiting ING5-mediated activation of PI3K/Akt pathway. Notably, UA could also reverse sorafenib resistance of HCC cells by suppressing the ING5-ACC1/ACLY-lipid droplets (LDs) axis. UA abrogated ING5 transcription and downregulated its expression by reducing SRF and YY1 expression and the SRF-YY1 complex formation. Alb/JCPyV T antigen mice were used for *in vivo* experiments since T antigen upregulated ING5 expression by inhibiting the ubiquitin-mediated degradation and promoting the T antigen-SRF-YY1-ING5 complex-associated transcription. UA suppressed JCPyV T antigen-induced spontaneous HCC through inhibiting ING5-mediated PI3K/Akt signaling pathway. These findings suggest that UA has the dual antitumoral functions of inhibiting hepatocellular carcinogenesis and reversing sorafenib resistance of HCC cells through targeting ING5, which could serve as a potential therapeutic strategy for HCC.

## Introduction

Hepatocellular carcinoma (HCC) still accounts for the leading cause of cancer-related mortality worldwide 1, 2. Due to its insidious onset and limitations in early detection, over half of HCC cases present advanced and inoperable disease 3. Despite recent groundbreaking progress in systemic therapy for advanced HCC, drug resistance and adverse reactions substantially affect its therapy effects 4. Therefore, the need to explore novel therapeutic targets and identify new drugs for HCC is urgent.

Ursolic acid (UA) is a pentacyclic triterpenoid compound extracted from Chinese herbs, such as *Hedyotic diffusa*, *Ligustrum lucidum*, and *Crataegus pinnatifida*, that has anti-inflammatory, antioxidant, antiviral, and hepatoprotective activities, as well as impressive antitumor effects on prostate cancer, breast cancer, and colorectal cancer 5-7. UA might inhibit cholesterol biosynthesis to induce cell cycle arrest and apoptosis in HCC cells 8. Moreover, UA could suppress the growth of HCC cells via Stat3 pathway or AMPKα-mediated reduction of DNA methyltransferase 1 9, 10. In one study, in cisplatin-resistant HepG2/cisplatin cells, UA targeted the Nrf2/antioxidant response element pathway to reverse cisplatin resistance 11. The comprehensive and precise clarification of antitumor mechanisms of UA would be helpful for its application.

The inhibitor of growth (ING) family, an epigenetic reader of the H3K4me3 histone, participates in the formation of histone acetyltransferase (HAT) and histone deacetylase (HDAC) complexes 12, 13. Among them, ING5 possesses a leucine zipper-like, a novel conserved region, a nuclear localization signal and a plant homeodomain (PHD) 12. Through its PHD, ING5 can promote histone H3 and H4 acetylation by via HBO1 or and the MOZ/MORF complex, respectively 12, 13. Generally, proteins acetylated by ING5 exert transcription cofactor and chromatin binding functions in the nucleus, while mediating cell metabolism in the cytoplasm 12, 13. Reportedly, ING5 suppressed the aggressiveness of prostate cancer by inhibiting Akt and inducing p53 signaling 14. Moreover, ING5 overexpression inhibited colorectal cancer progression via inactivation of PI3K/Akt pathway 15. In contrast, ING5 also enhanced the self-renewal of glioblastoma stem cells 16 and induced the chemoresistance of colorectal cancer cells according to our unpublished data. In the present study, we demonstrated that UA inhibited HCC progression by disturbing ING5-mediated PI3K/Akt signaling pathway. Meanwhile, UA reduced the binding of serum response factor (SRF) and Yin Yang-1 (YY1) to the promoter of ING5, thereby downregulating the expression of ING5. Moreover, UA interrupted lipogenesis by inhibiting ING5 expression and reversed the chemoresistance of HCC cells to sorafenib.

## Materials and Methods

### Cell culture and reagents

The human HCC cell lines HepG2, PLC/PRF/5, Huh7, MHCC97-H and Hep3B, the mouse HCC cell line Hepa1-6, and the human immortalized liver cell line THLE-2 were purchased from the Chinese Academy of Sciences, Cyagen Biosciences or CTCC, and cultured in MEM, DMEM or BEGM (Corning, Manassas, VA, USA) supplemented with 10% fetal bovine serum (FBS, Cell-Box, Australia). All cells were cultured in a humidified incubator with 5% CO_2_ at 37 °C. All cell lines were confirmed to be free of mycoplasma contamination. Sorafenib-resistant HepG2 cells were established with sorafenib concentrations gradually increasing from 1 μM to the maximum tolerated dose, and ultimately maintained in the medium mentioned above containing 2 μM sorafenib. Primary HCC cells were isolated from the spontaneous HCC of Alb-cre/JCPyV transgenic mice as previously described 17 and maintained in DMEM containing 10% FBS. Ursolic acid (UA, U6753) was purchased from Sigma-Aldrich/Merck (St. Louis, MO, USA); sorafenib(S7397) from Selleck Chemicals (Shanghai, China); PI3K inhibitor LY294002 (S1737) from Beyotime (Shanghai, China); ATP-citrate lyase (ACLY) inhibitor (M5207); acetyl-CoA carboxylase 1 (ACC1) inhibitor PF-05175157 (M6137) and proteasomal inhibitor MG132 (M1902) from Abmole (Houston, TX, USA), and cycloheximide (CHX) (HY-12320) from MCE (MedChemExpress, Shanghai, China).

### Cell transfection

ACC1, ACLY and T antigen overexpression and shRNA plasmids (HonorGene, Changsha, China) and SRF (sc-36563, Santa Cruz, CA, USA) and YY1 (sc-36863, Santa Cruz, CA, USA) siRNA plasmids were transfected into cells using a Lipofectamine 3000 transfection kit (L3000015, Invitrogen, USA). ING5 lentiviral overexpression and shRNA plasmids were constructed by Obio Technology (Shanghai, China), and transfected into cells as per the manufacturer's protocol. The stably-expressing cells were screened with puromycin (58-58-2, Solarbio, Beijing, China) or G-418 disulfate (108321-42-2, Solarbio, Beijing, China), and verified by Western blot or quantitative real-time PCR (qRT‒PCR).

### Cell proliferation

A Cell Counting Kit-8 (CCK-8) kit (CT01A, Cellcook Biotech, Guangzhou, China) was employed to assess cell viability/cytotoxicity. Briefly, cells were seeded into 96-well plates. Following treatment, each well was replaced by 100 μl fresh medium containing 10 μl of CCK-8 reagent and incubated at 37 °C for 2 h. The absorbance was then detected at 450 nm by a Multiskan™ FC Microplate Photometer (Thermo Fisher Scientific). For the colony formation assay, cells were seeded in 6-well plates at a density of 500 cells/well and cultured overnight. After treatment, the medium was changed every 3 days until the cell colonies were visible. Then, these colonies were methanol-fixed, stained with 0.5% crystal violet and counted. Additionally, a BeyoClick™ EdU Cell Proliferation Kit with Alexa Fluor 555 (C0075L, Beyotime, Shanghai, China) was used to assess cell proliferation.

### Flow cytometry assay

Cell cycle, apoptosis and mitochondrial membrane potential (MMP) assays were performed by flow cytometry (Beckman Coulter, Brea, CA, USA) using the following: Cell Cycle Detection Kit (KGA512, KeyGEN BioTECH, Nanjing, China), FITC Annexin V Apoptosis Detection Kit I (556547, BD Biosciences, USA), Annexin V-PE/7-AAD Apoptosis Detection Kit (KGA1018, KeyGEN BioTECH, Nanjing, China) or JC-10 MMP Assay Kit (CA1310, Solarbio, Beijing, China).

### Wound healing assay

Briefly, cells were seeded in 6-well plates, reaching 80% confluence and then carefully scratched with a sterile 200 μL pipette tip. These cells were cultured in serum-free medium following three washes with PBS. Finally, the wounds were monitored, and photographed, and measured using ImageJ software (NIH, USA).

### Transwell assay

Transwell chambers (3422, Costar, Corning, USA) precoated with Matrigel® Basement membrane matrix (356234, Corning Life Sciences, MA, USA) were used for cell invasion assays. First, 600 μL of medium with 15% FBS was added to the lower chamber, and cells with indicated treatments in 200 μL medium with 2% FBS were added to the upper chamber. After 24-48 h of incubation at 37 °C, the cells on the lower surface of the chamber membrane were fixed in 100% ethanol, stained with 0.5% crystal violet, and counted under a microscope.

### Nile red staining

Cells were fixed in in 100% methanol for 10 min following three washes with PBS. Next, cells were incubated with Nile red staining solution (1 mg/ml) for 5 min after rinsing with PBS three times. Cells were imaged using an inverted fluorescence microscope following counterstaining with DAPI (C1006, Beyotime, Shanghai, China) for 10 min. Finally, images were analyzed using ImageJ software.

### Western blot

Protein was extracted from cell or tissue lysates with RIPA lysis buffer (P0013D, Beyotime, Shanghai, China) with freshly added protease and phosphatase inhibitor cocktail (P1046, Beyotime, Shanghai, China). Protein concentrations were determined with a Bicin-choninic Acid Protein Assay Kit (CW0014, Cowin Biotech Co., Jiangsu, China). The equal amount of protein samples were resolved by SDS-PAGE and transferred onto PVDF membranes (GVS Life Science). After blocking for 1 h at room temperature in 5% skim milk, the membranes were incubated at 4 °C overnight with primary antibodies (Table S1). The following day, the membranes were incubated with horseradish peroxidase-labeled IgG antibodies, developed with an enhanced chemiluminescence kit (NCM Biotech, Newport, RI, USA; Cat. No. P10300), and then exposed using an Azure Biosystem C300.

### qRT‒PCR

Total RNA was extracted with TRIzol reagent (15596026, Invitrogen, USA) and was then reverse-transcribed into cDNA using a FastKing RT Kit (KR116, Tiangen Biotech, Beijing, China) according to the manufacturer's protocol. NovoStart SYBR qPCR SuperMix Plus (E096-01A, Novoprotein Scientific, Jiangsu, China) was used to conduct qRT‒PCR in the ABI PRISM 7900 Sequence Detection System (Applied Biosystems). The primers used were shown in Table S2. The relative expression levels of genes were normalized to GAPDH and calculated with the 2^-ΔΔCt^ method.

### Co‒immunoprecipitation (Co‒IP)

After washing in PBS, cells were lysed in ice-cold lysis buffer. Then, 1.0 μg of primary antibody or appropriate control IgG was added to aliquots of 400 μg of cellular protein for 1 h at 4 °C. After that, each immunoprecipitation mixture was precipitated by adding 20 μl of Protein A/G PLUS-Agarose (sc2003, Santa Cruz Biotechnology, CA, USA) for 2 h at 4 °C on a rocker platform. After washing 4 times with lysis buffer, the immunoprecipitates were boiled in SDS-PAGE loading buffer for subsequent Western blotting.

### Chromatin immunoprecipitation (ChIP) assay

1 × 10^7^ cells were incubated for 10 min at room temperature in 1% paraformaldehyde. The following steps were performed with the SimpleChIP Plus Sonication Chromatin IP Kit (56383, Cell Signaling Technology, Danvers, MA, USA). 10 μg of chromatin fragments were immunoprecipitated with SRF, YY1, histone H3 (positive control), or normal rabbit IgG (negative control) antibody at 4 °C with rotation overnight. Finally, the purified DNA fragments were analyzed through agarose electrophoresis and qRT‒PCR. The primers used for detecting the SRF and YY1 binding regions in the ING5 promoter were as follows: P1F (5'-gcatgcatcttacggcacac-3') and P1R (5'-gccacctctcgaggcagg-3'), P2F (5'-cgcgcgactcatgaatagtg-3') and P2R (5'-agtgctccaagtacatggcg-3'), respectively.

### Dual-luciferase reporter assay

ING5 promoter sequences containing the wild-type (WT) or mutant (MUT) binding site of SRF or YY1 were constructed and cloned into the pGL3-basic plasmid (Promega, Madison, USA) by ProbeGene (Jiangsu, China). The ING5 promoter activity was evaluated as previously described 18.

### Protein stability assay

To characterize protein stability, cells were first treated with 0.5 μg/ml CHX to stop *de novo* protein synthesis. The cells were harvested at specific time points, and the protein expression levels were analyzed by Western blotting.

### Animal experiments

Four mice were housed in each plastic cage with paper chips. All had access to standard rodent food (Beijing HFK Bioscience) and water, and were housed in a temperature-controlled animal room with a 12-h light/dark cycle. The animal procedures used were performed in accordance with the Guide for the Care and Use of Laboratory Animals and approved by the Committee on Animal Experimentation of the Affiliated Hospital of Chengde Medical University (No. CYFYLL2022094). Alb/JCPyV T antigen mice were successfully generated by mating CAG-loxp-LacZ T antigen mice with Alb-cre mice, as demonstrated in our previous studies 17. Male mice, which exhibited a notably higher incidence of hepatic tumors than female mice, developed HCC beginning at approximately 12 weeks of age, with a 100% of tumor incidence at 24 weeks of age (Table S3). Therefore, male Alb/JCPyV T antigen mice were prepared for the experiments. To investigate the tumor-suppressive effect of UA on HCC *in vivo*, mice were administered vehicle, 50 mg/kg, or 100 mg/kg UA via oral gavage twice a week from 18 to 30 weeks of age. Ultrasonography was performed at 22, 24, 26, 28, and 30 weeks of age. Mice were sacrificed by cervical dislocation following the anesthetization with inhaled isoflurane (1-2%), and samples were collected at 32 weeks of age.

### PCR

DNA for genotyping DNA was obtained from mouse tails by precipitation with two volumes of ethanol following proteinase K digestion. Then, the genomic DNA was amplified by PCR using 2×Taq Plus Master Mix (Dye) (CW2849H, CoWin Biotec, Beijing, China) and the following primers: JCPyV T antigen (Forward: 5'-TGGCCTGTAAAGTTCTAGGCA-3' and Reverse: 5'-GCAGAGTCAAGGGATTTACCTTC-3'), Alb-cre (Forward: 5'-GCCTGCATTACCGGTCGATGC-3' and Reverse: 5'-CAGGGTGTTATAAGCAATCCC-3'). The PCR products were electrophoresed in a 1% agarose gel, and imaged using imaging system (Azure Biosystem C200) for genotyping.

### Ultrasonography

The ultrasound examination was performed with a Mindray MX7 device (Shenzhen Mindray Bio-medical Electronics, Shenzhen, China) by the same expert ultrasonologist. Mice were anesthetized with inhalation isoflurane (1-2%) and then kept on a homeothermic pad during the operation. The abdominal area was coated with ultrasound gel after depilation using depilatory cream for improved ultrasound imaging.

### Histopathology

Mouse liver tissues were fixed in 4% paraformaldehyde solution for 24 h, embedded in paraffin, and sectioned into 4-μm slices. For histopathological diagnoses of HCC, liver slices were routinely stained with hematoxylin and eosin (HE). Immunohistochemistry (IHC) was performed as previously described 19. Apoptosis in mouse tissue sections was assessed by terminal deoxynucleotidyl transferase dUTP nick end labeling (TUNEL) assay using a TUNEL Apoptosis Assay Kit (KGA703, KeyGEN Biotech, Jiangsu, China). The apoptosis index was measured as the percentage of TUNEL-positive cells in 4/5 random visual fields per tissue section.

### Enzyme-linked immunosorbent assay (ELISA)

Mouse plasma was collected by centrifuging blood samples at 3000 ×g for 10 min. Liver function in indicators, including alanine aminotransferase (ALT) and aspartate aminotransferase (AST), in the serum were measured using ELISA kits following the manufacturer's instructions (JL12668 and JL13992, Jianglai Biotech, Shanghai, China).

### Bioinformatics analysis

The expression of ING5, SRF, and YY1 in normal liver tissues and HCC tissues was analyzed in UALCAN (http://ualcan.path.uab.edu), Human Protein Atlas (HPA) repository (http://www.proteinatlas.org/) and Gene Expression Omnibus (GEO) database (https://www.ncbi.nlm.nih.gov/geo/). The clinicopathological and prognostic significance of ING5, SRF, and YY1 mRNA expression in HCC patients were explored using the Xiantao platform (https://www.xiantaozi.com/) and Kaplan-Meier (KM) plotter (http://kmplot.com/). Correlation analysis of mRNA expression was performed with R software following RNA-sequencing expression profile data downloaded from the TCGA dataset (https://portal.gdc.com).

### Statistical analysis

All statistical analyses were done using SPSS software (version 26, IBM Corp., USA) and GraphPad Prism software (version 9.0, GraphPad Software Inc., USA). Student's t test and one-way analysis of variance (ANOVA) were used for comparisons between two groups and among multiple groups, respectively. All data were shown as the mean ± standard deviation (SD) unless indicated otherwise. P value < 0.05 was considered as statistical significance (n.s.=not significant, *P < 0.05, **P < 0.01, ***P < 0.001).

## Results

### UA inhibited the aggressive phenotypes of HCC cells

**Figure 1:**
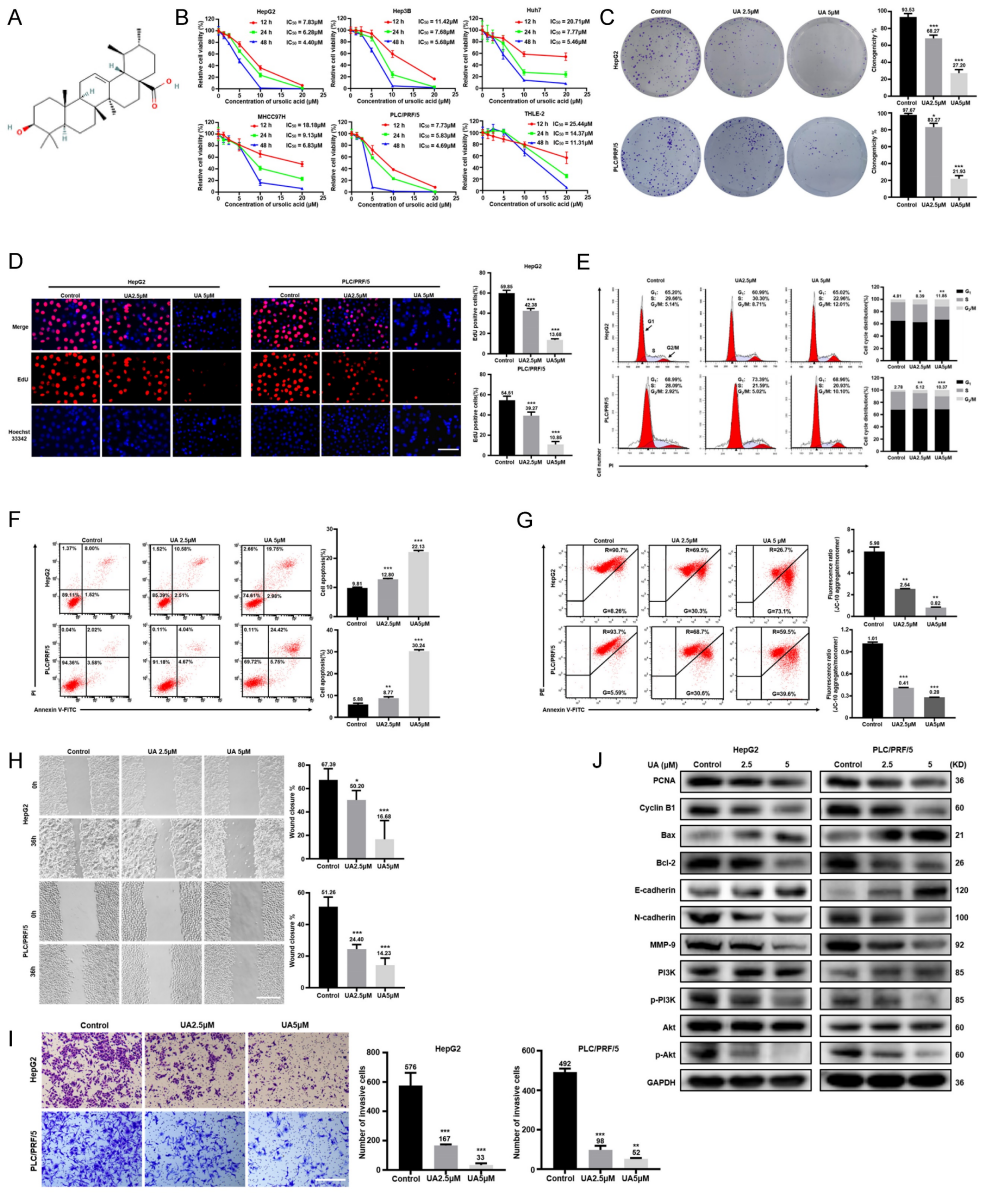
** UA inhibited the malignant biological behaviors of hepatocellular carcinoma cells.** (A) Chemical structure of available ursolic acid (UA) (PubChem CID: 64945, https://pubchem.ncbi.nlm.nih.gov/compound/Ursolic-Acid). (B) Various hepatocellular carcinoma (HCC) cells and human normal hepatocytes THLE-2 were treated with UA (0, 1.25, 2.5, 5, 10, and 20 μM) for the indicated times (12, 24, and 48 h) and subjected to CCK-8 assays. We treated HepG2 and PLC/PRF/5 cells with 2.5 and 5 μM UA for 24 h and performed colony formation (C) (n=3), EdU incorporation (D) (Scale bar =30μm, n=5), cell cycle (E) (n=3), apotosis (F) (n=3) and MMP (G) (n=3) assays. Wound healing (H) (scale bar =50μm, n=5) and Transwell(I) (scale bar =50 μm, n=3) assays were performed to determine the effects of UA on the migration and invasion of HCC cells. The expression of phenotype-related proteins was determined by western blot (J). All data are presented as mean ± SD, and Student's t test was used for significance tests, *P < 0.05, **P < 0.01, ***P < 0.001 vs. the control group. UA, ursolic acid. MMP, mitochondrial membrane potential.

As shown in Fig. 1B, UA significantly inhibited the cell viability of HepG2, Hep3B, Huh7, MHCC97H, and PLC/PRF/5 cells in a dose- and time-dependent manners by CCK-8 assays, while human normal hepatocytes THLE-2 showed a less inhibition of viability of with UA treatment than HCC cell lines. UA inhibited the viability of HepG2 and PLC/PRF/5 cells more potently than other HCC cells. Thus, HepG2 and PLC/PRF/5 cells were selected for subsequent experiments. Based on the IC50 value of HepG2 (6.28μM) and PLC/PRF/5 cells (5.83μM) at 24 h, the UA concentrations of 2.5 and 5.0μM were used as non-toxic doses. According to colony-formation, EdU incorporation and cell cycle assays, UA markedly reduced the clonogenicity and the percentage of EdU-positive cells and arrested G_2_/M in HCC cells (Fig. 1C-E). UA substantially augmented the apoptotic ratio of HCC cells (Fig. 1F) and reduced the MMP (Fig. 1G), which is known to induce the mitochondrial caspase-dependent apoptosis pathway. The migration and invasion of both HCC cells treated with UA for 36h were significantly suppressed in a concentration-dependent manner, evidenced by wound healing and Transwell assays (Fig. 1H and 1I). Western blot analysis showed that UA treatment attenuated the expression of PCNA, Cyclin B1, MMP-9, N-cadherin, Bcl-2, PI3K, p-PI3K, Akt and p-Akt, but increased the expression of Bax and E-cadherin in a concentration-dependent manner (Fig. 1J).

### UA suppressed the malignant progression of HCC via NG5 downregulation

**Figure 2:**
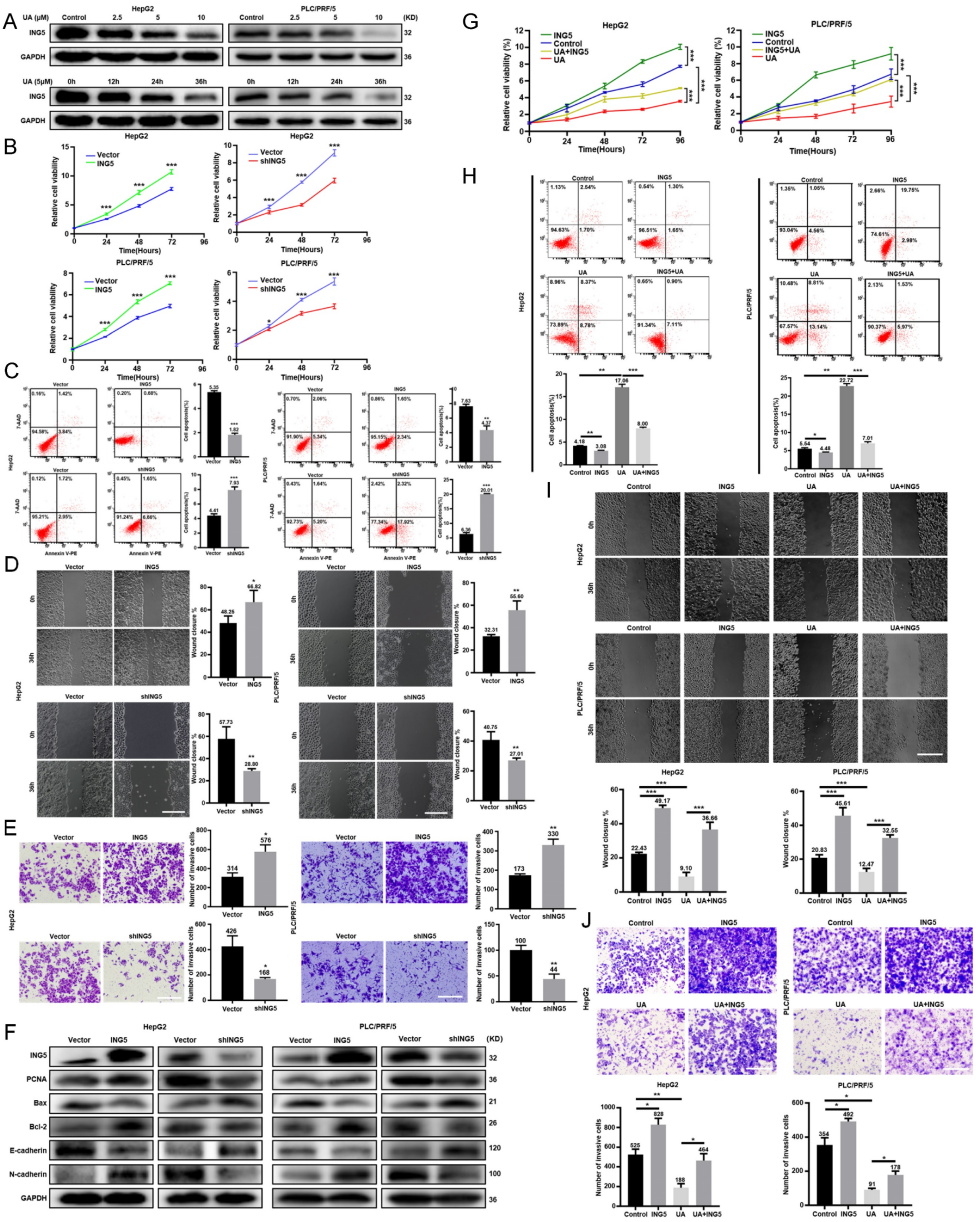
** UA suppressed the malignant progression of hepatocellular carcinoma via the downregulation of ING5.** (A) HepG2 and PLC/PRF/5 cells were treated with UA (0, 2.5, 5, and 10 μM) for 12, 24, and 36 h and subjected to Western blot analysis for ING5 expression. The effects of ING5 expression on HCC cells viability, apoptosis, migration, and invasion were measured by CCK-8 (B), flow cytometry (C) (n=3), wound healing (D) (n=5), and Transwell assays (E) (n=3), respectively (*P < 0.05, **P < 0.01, ***P < 0.001 vs. the vector group. Scale bar =50 μm). (F) Phenotype-associated proteins were examined by western blot. Cell viability (G), apoptosis (H) (n=3), migration (I) (n=5), and invasion (J) (n=3) were analyzed in HepG2 and PLC/PRF/5 cells after overexpressing ING5 or/and treatment with 5 μM UA (*P < 0.05, **P < 0.01, ***P < 0.001. Scale bar =50 μm). All data are presented as mean ± SD, Student's t test was used for the significance tests. UA, ursolic acid.

UA treatment decreased ING5 expression in HepG2 and PLC/PRF/5 cells in a concentration- and time-dependent manner by western blotting (Fig. 2A). According to the HPA database, ING5 was localized in the cytoplasm and nucleus and showed a higher expression in HCC than normal liver tissues (Fig. S1A). Furthermore, LIHC and CPTAC data from TCGA, GSE102079 and GSE76427 datasets from GEO databases showed that ING5 mRNA and protein expression levels was elevated in HCC tissue in comparison to normal tissue (Fig. S1B). According to TCGA (LIHC-TCGA), ING5 mRNA expression was positively correlated with tumor status, a high level of serum alpha-fetoprotein (AFP) (>400 ng/ml), vascular invasion, and histologic grade (Table S4). The KM plotter database showed that ING5 mRNA expression was positively correlated with poor overall survival (OS), progression-free survival (PFS), relapse-free survival (RFS), and disease-specific survival (DSS) of HCC patients (Fig. S1C and Table S5). In addition, high ING5 expression was also associated with poor OS of HCC patients with stage 2-3, 3, grade 3, T2 and T3, poor PFS of stage 2, 2-3, 3, 3-4, grade 1, T2 as well as short RFS of stage 2, 2-3, grade 1, T2 (Table S5).

We silenced or overexpressed ING5 in HepG2 and PLC/PRF/5 cells separately, evidenced by western blot analysis (Fig. S1D) and qRT‒PCR (Fig. S1E). ING5 overexpression significantly increased viability HCC cells (Fig. 2B), migration (Fig. 2D) and invasion (Fig. 2E) of HCC cells, compared to the vector control. Annexin V-PE /7-AAD staining showed that ING5 overexpression decreased apoptosis in comparison to the vector control (Fig. 2C). Western blot analysis showed that ING5 increased the expression of PCNA, Bcl-2, N-cadherin and decreased the expression of Bax, E-cadherin (Fig. 2F). Conversely, ING5 knockdown exerted the opposite effects (Fig. 2B-F). Intriguingly, ING5 overexpression attenuated these effects of UA at the same concentration (Fig. 2G-J). And ING5 silencing also weakened these effects induced by UA treatment (Fig. S2A-D).

### UA inhibited HCC progression by disturbing ING5-mediated PI3K/Akt signaling pathway

**Figure 3:**
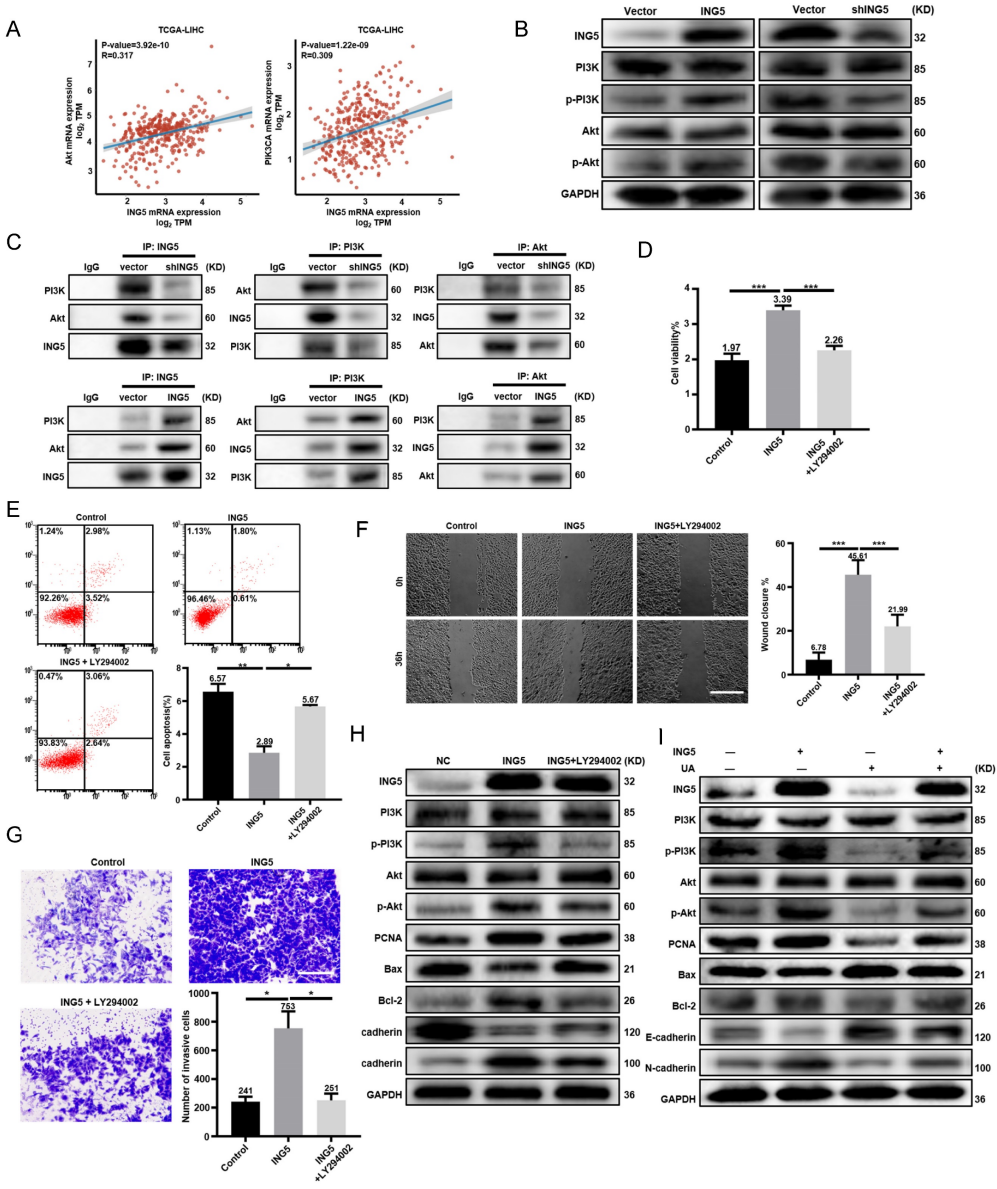
**UA inhibited hepatocellular carcinoma progression by disturbing the ING5-mediated PI3K/Akt signaling pathway.** (A) The correlations between ING5 mRNA and Akt or PIK3CA mRNA were analyzed using the ggplot2 library in R software. (B) PI3K/Akt signaling pathway related proteins in ING5-overexpressing or -silencing HepG2 cells were screened by western blot. (C) Co-IP was performed to assess the interaction between ING5 and PI3K/Akt. HepG2 cells overexpressing ING5 were treated with 10 μM LY294002 for 24 h, and cell viability, apoptosis, migration, and invasion were measured by CCK-8 (D), Annexin V staining (E) (n=3), wound healing (F) (n=5), and Transwell assays (G) (n=3), respectively. (H) Western blot assays were performed to determine the expression of proteins related to the PI3K/Akt signaling pathway and phenotype-related proteins after ING5-overexpressing HepG2 cells with 10μM LY294002 treatment for 24 h. (I) Western blot analyses were performed to confirm that treatment with 5 μM UA attenuated the ING5-induced changes in the phosphorylation of PI3K and Akt, and the expressions of phenotype-related proteins in HepG2 cells. All data are presented as mean ± SD, and Student's t test was used for significance tests, *P < 0.05, **P < 0.01, ***P < 0.001. Scale bar = 50μm. UA, ursolic acid. IP, immunoprecipitation.

There existed positive associations of ING5 with PI3K and Akt mRNA expression in HCC according to TCGA-LIHC database (Fig. 3A). Western blot analysis showed that ING5 knockdown decreased the phosphorylation of PI3K and Akt in HepG2 cells. As shown in Fig. 3C, ING5 knockdown reduced the interaction of ING5 with PI3K and Akt. In contrast, ING5 overexpression had the opposite results (Fig. 3B-3C). PI3K inhibitor LY294002 (10 μM) significantly inhibited the cell viability (Fig. 3D), anti-apoptosis (Fig. 3E), migration (Fig. 3F), and invasion (Fig. 3G) of ING5 transfectants by CCK-8, Annexin V staining, wound healing and Transwell assays, respectively. Furthermore, western blot analysis indicated that LY294002 reduced the phosphorylation of PI3K and Akt, and the expression of PCNA, Bcl-2 and N-cadherin, and increased Bax and E-cadherin expression in ING5 transfectants (Fig. 3H). Although ING5 overexpression significantly upregulated the phosphorylation of PI3K and Akt, and PCNA as well as Bcl-2/Bax, N-cadherin/E-cadherin ratio, UA treatment significantly alleviated the effect of ING5 on HepG2 cells (Fig. 3I).

### UA reversed sorafenib resistance in HCC cells by inhibiting ING5-mediated lipogenesis

**Figure 4:**
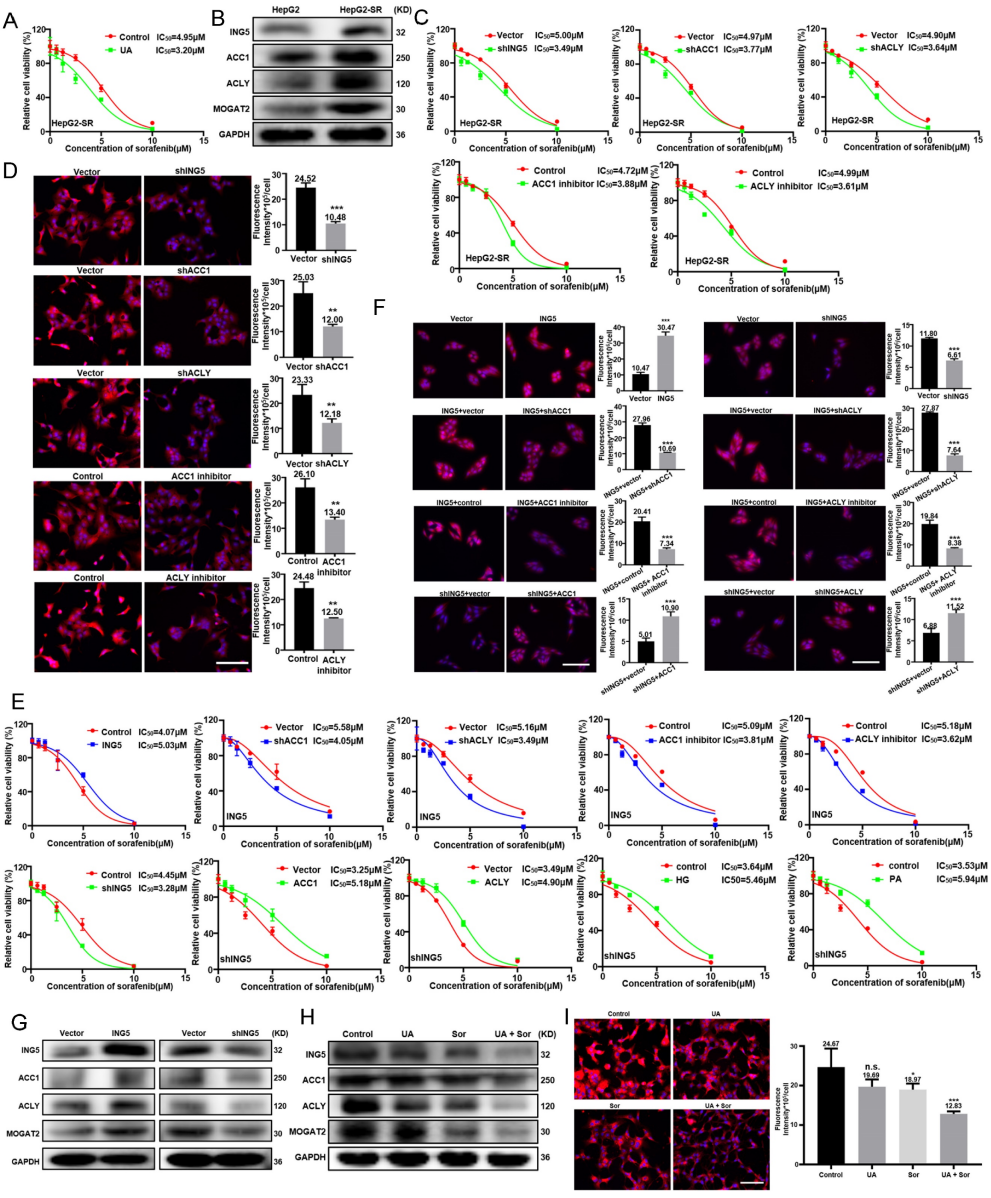
** UA reversed sorafenib resistance in hepatocellular carcinoma cells by inhibiting ING5-mediated lipogenesis.** (A) After treatment with sorafenib alone or in combination with 3 μM UA for 24 h, cell viability was analyzed by CCK-8 assays. (B) Western blotting was performed to assess the expression of ING5 and lipogenic enzymes (ACC1, ACLY, and MOGAT2). Following the silencing of ING5, ACC1 or ACLY expression, or treatment with 10 μM ACC1 or ACLY inhibitor for 24 h, HepG2-SR cells were subjected to the CCK-8 assay (C) and Nile red staining (D). The viability of ING5-overexpressing or silencing HepG2 cells was measured by CCK-8 assays (E) and Nile red staining (F) after treatment with sorafenib for 24 h. ING5-overexpressing HepG2 cells were treated with transfection of sh-ACC1 or -ACLY, or exposure to 10μM ACC1 or ACLY inhibitor, whereas ING5-silencing HepG2 cells overexpressing -ACC1 or -ACLY, or exposure to 5 g/L HG or 50μM PA for 24 h. Then, the cell viability was analyzed by CCK-8 assay (E) and Nile red staining (F) after sorafenib treatment. (G) Western blotting was performed to explore the effect of ING5 on the expression of ACC1, ACLY and MOGAT2 in HepG2 cells. HepG2-SR cells were treated with 2 μM sorafenib alone or in combination with 3 μM UA for 24 h and subjected to western blotting (H) and Nile red staining (I). The data are presented as mean ± SD, and Student's t test was used for significance tests. *P < 0.05, **P < 0.01, ***P < 0.001 vs. the control group. Scale bar =30 μm. UA, ursolic acid. HG, high glucose. PA, palmitic acid. Sor, sorafenib.

We established a sorafenib resistant HepG2 cells, named HepG2-SR (Fig. S3A). Considering the IC50 (8.3 M) of UA on HepG2-SR cells, 3 μM UA was combined with sorafenib to treat HepG2-SR cells for 24 h (Fig. S3B). The IC50 of sorafenib decreased markedly from 4.95 μM to 3.20μM in HepG2-SR cells with 3 μM UA (Fig. 4A). HepG2-SR cells showed a higher expression of ING5, ACC1, ACLY, and MOGAT2 than the parental cells (Fig. 4B). The knockdown of ING5, ACC1 and ACLY, or treatment with ACC1 and ACLY inhibitors significantly reversed the insensitivity of HepG2-SR cells to sorafenib (Fig.4C and S3C), while reduced lipid droplets (LDs) formation in HepG2-SR cells (Fig. 4D). ING5 overexpression triggered sorafenib resistance of HepG2 cells, whereas its knockdown increase sensitivity to sorafenib in HepG2 cells (Fig. 4E). Interestingly, ACC1 and ACLY overexpression, or high glucose (HG) and palmitic acid (PA) treatment reduced the sorafenib-sensitizing effect of ING5 silencing. Likewise, ACC1 and ACLY silencing, or ACC1 and ACLY inhibitor reversed the sorafenib resistance of ING5 transfectants (Fig. S2C, Fig. 4E). Nile red staining showed that ING5 overexpression promoted LDs formation of HepG2 cells, but this effect was abated by ACC1 or ACLY silencing and inhibitor (Fig. 4F). The reduced LDs formation caused by knockdown of ING5 was rescued after upregulating ACC1 or ACLY expression (Fig. 4F). ING5 overexpression increased ACC1, ACLY, and MOGAT2 expressions, whereas its silencing exerted the opposite effect (Fig. 4G). The combination with UA decreased ING5, ACC1, ACLY and MOGAT2 expression, and thereafter reduced LDs formation more markedly compared with the sorafenib treatment in HepG2-SR cells (Fig. 4H and 4I).

### UA abrogated ING5 transcription in HCC by downregulating SRF and YY1 expression and disassociating the SRF-YY1 complex

**Figure 5:**
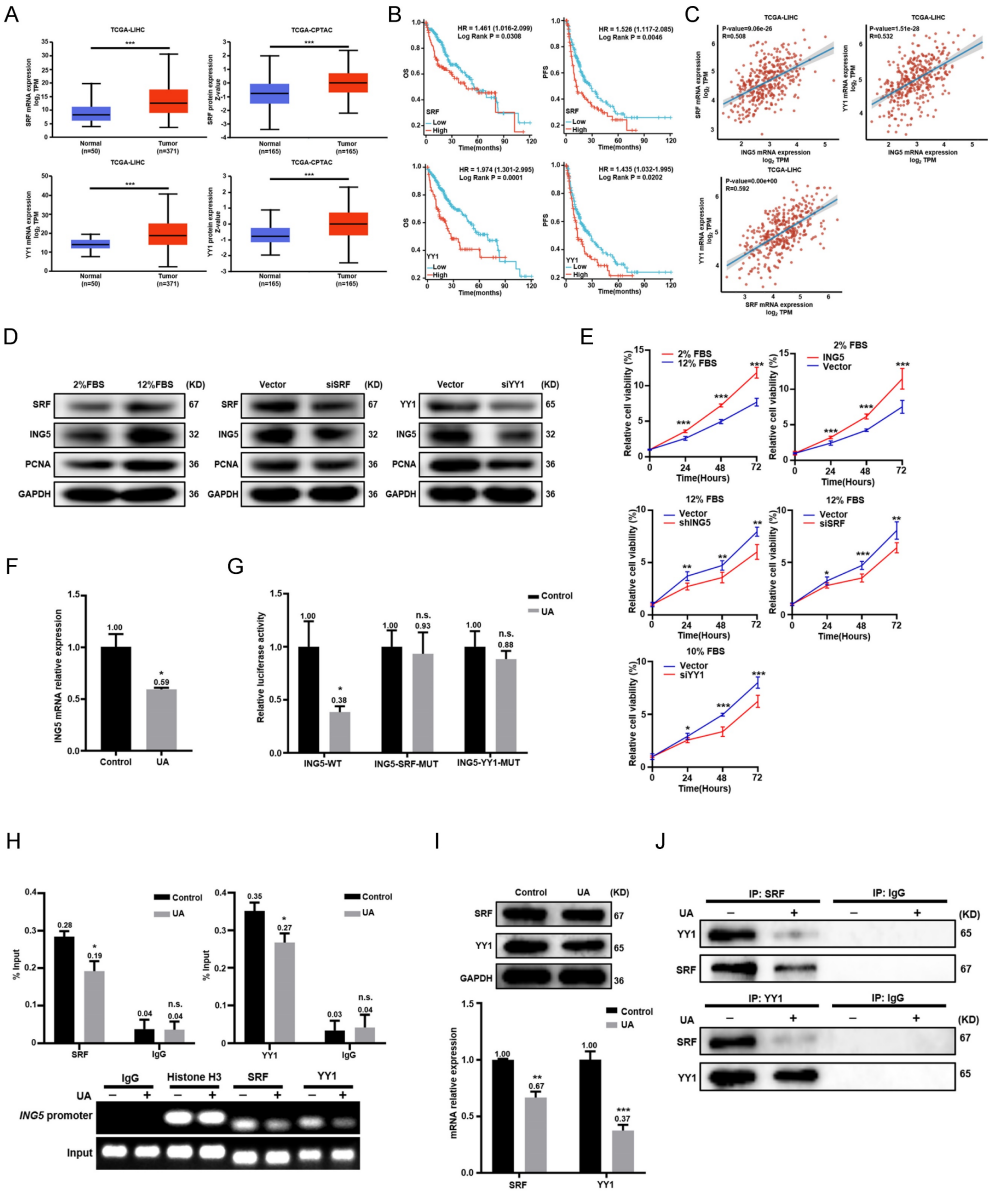
** UA abrogated ING5 transcription in hepatocellular carcinoma cells by reducing SRF and YY1 expression and disassociating the SRF-YY1 complex.** (A) SRF and YY1 expressions was analyzed in hepatocellular carcinoma (HCC) and normal liver tissues using TCGA-LIHC and TCGA-CPTAC data from UALCAN (***P < 0.001). (B) The prognostic significance of SRF and YY1 mRNA expression in HCC was studied using Xiantao (https://www.xiantaozi.com/) (HR, hazard ratio). (C) The correlations among SRF, YY1 and ING5 were assessed by R software. (D) HepG2 cells were exposed to the culture medium containing 2% and 12% FBS for 36 h, respectively (left). HepG2 cells were transfected with SRF siRNA for 24 h following exposure to medium containing 12% FBS for 36 h (middle). HepG2 cells were transfected with YY1 siRNA for 24 h following exposure to the medium containing 12% FBS for 36 h (right). The expression levels of SRF, ING5 and PCNA were determined by western blotting. (E) HepG2 cells were exposed to the culture medium containing 2% and 12% FBS for 36 h. HepG2 cells were transfected with ING5 shRNA, SRF or YY1 siRNA following exposure to the medium containing 12% FBS for 36 h. HepG2 cells were transfected with ING5 overexpression plasmid following exposure to the medium containing 2% FBS for 36 h. CCK-8 assays were performed for assessing the proliferation of HepG2 cells (n=3, *P < 0.05, **P < 0.01, ***P < 0.001). (F) qRT‒PCR assays were performed to assess the expression of ING5 mRNA in HepG2 cells treated with 5 μM UA for 24 h (n=3, *P < 0.05 vs. the control group). (G) HepG2 cells were individually transfected with the pGL3-basic reporter vector containing the WT or MUT SRF/YY1 binding site in the ING5 promoter sequence, treated with 5 μM UA for 24 h, and subjected to the dual-luciferase reporter assay (n=3, n.s.=not significant, *P < 0.05 vs. the control group). (H) At 24 h after UA treatment, ChIP assays with anti-SRF and anti-YY1 antibodies were used to measure the bindings of SRF and YY1 to the promoter of ING5 in HepG2 cells (n=3, n.s.=not significant, *P < 0.05 vs. control group). (I) The expression levels of SRF and YY1 in HepG2 cells were measured after the exposure to 5 μM UA for 24 h by western bloting (upper) and qRT‒PCR (lower) (n=3, **P < 0.05, ***P < 0.001 vs. control group). (J) Co-IP assays with anti-SRF and anti-YY1 antibodies were performed to assess the binding between SRF protein and YY1 protein in HepG2 cells with 5 μM UA for 24 h (IP, immunoprecipitation). All data are presented as mean ± SD, Student's t test was used for the significance tests. UA, ursolic acid.

According to TCGA data from UALCAN, the expression of SRF or YY1 was higher in HCC than in normal liver tissues at both RNA and protein levels (Fig. 5A). Both SRF and YY1 mRNA expressions were negatively associated with OS and PFS of HCC patients by Xiantao platform (Fig. 5B). The significant positive correlations existed between SRF, YY1 and ING5 mRNA expression in HCC (Fig. 5C). Given that SRF was highly serum inducible, high FBS (12% FBS) increased the expression of SRF, ING5 and PCNA in HepG2 cells. However, SRF silencing resulted in the decrease of ING5 and PCNA expression (Fig. 5D). Additionally, high FBS significantly promoted the proliferation and clonogenicity of HepG2 cells, while silencing of SRF or ING5 abolished these effects (Fig. 5E and S3D). Furthermore, ING5 overexpression significantly increased the proliferation and clonogenicity of HepG2 cells exposed to low FBS (2% FBS) (Fig. 5E and S3D). We also transfected HepG2 cells with YY1 siRNA, and demonstrated that YY1 silencing decreased ING5 as well as PCNA expression, and inhibited the proliferation and clonogenicity in HepG2 cells (Fig. 5D-5E and S3D). In HepG2 cells, ING5 mRNA expression was significantly decreased after UA treatment (Fig. 5F). As shown in Fig. 5G, UA treatment (5 μM, 24 h) significantly diminished the promoter activity of WT pGL3-ING5 rather than MUT type in HepG2 cells. ChIP assays also showed that UA markedly reduced the binding of SRF and YY1 to the promoter of ING5 (Fig. 5H). There was a remarkable decrease in SRF and YY1 at either mRNA or protein level in HCC cells treated with UA (Fig. 5I). Notably, UA treatment suppressed SRF-YY1 complex formation in HepG2 cells after UA treatment, as shown by Co-IP (Fig. 5J).

### ING5 contributed to the carcinogenesis and progression of JCPyV T antigen-related HCC

**Figure 6:**
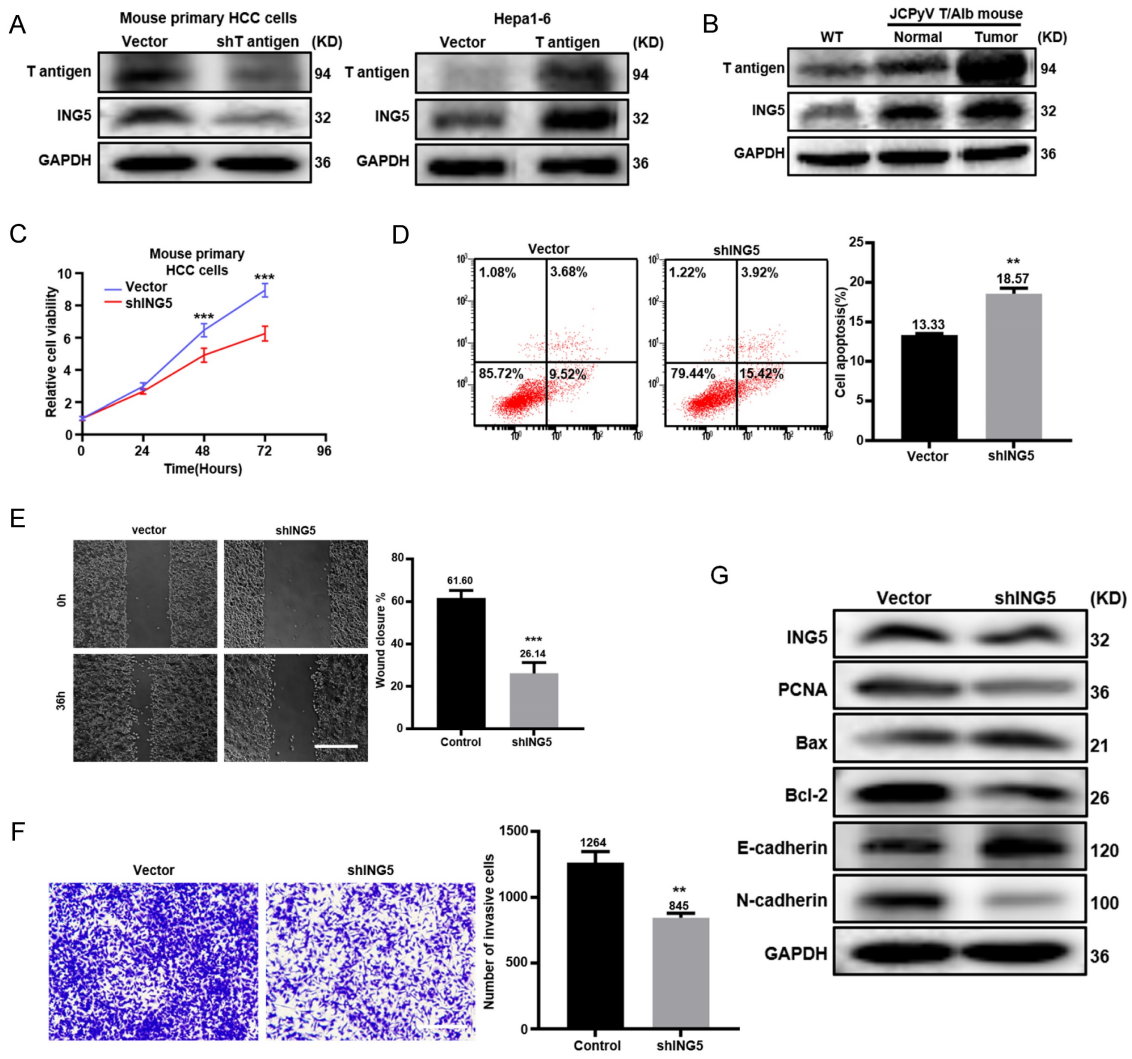
** ING5 contributed to the carcinogenesis and progression of JCPyV T antigen-related HCC.** (A) T antigen and ING5 protein levels were checked by Western blot after silencing T antigen in mouse primary hepatocellular carcinoma (HCC) cells (left) and overexpressing T antigen in mouse HCC cell line Hepa1-6 (right). (B) T antigen and ING5 protein expression was examined in normal liver tissues from wild-type (WT) mice, para-carcinoma and tumor tissues from Alb/JCPyV T antigen mice by Western blotting. Primary HCC cell from Alb/JCPyV T antigen transgenic mice were transfected with sh-ING5, and subjected to CCK-8 (C), Annexin V staining (D) (n=3), wound healing (E) (n=5), Transwell assays (F) (n=3) and western blot assays (G). All data are presented as mean ± SD, and Student's t test was used for the significance tests.**P < 0.01, ***P < 0.001 vs. the vector group. Scale bar = 50 μm. WT, wild-type.

Primary HCC cells from Alb/JCPyV T antigen transgenic mice were transfected to silence T antigen. Western blot analysis showed that T antigen knockdown decreased ING5 expression, while T antigen overexpression had the opposite results in mouse Hepa1-6 cells (Fig. 6A). T antigen and ING5 expression sequentially increased from normal liver tissue, para-carcinoma and tumor tissue from Alb/JCPyV T antigen transgenic mice (Fig. 6B). ING5 silencing in Alb/JCPyV T antigen transgenic mouse primary HCC cell reduced cells proliferation (Fig. 6C), migration (Fig. 6E), invasion (Fig. 6F) and promoted apoptosis (Fig. 6D). Western blot analysis confirmed that PCNA, Bcl-2 and N-cadherin expression was downregulated, and that Bax and E-cadherin expression was upregulated in shING5 transfectants of Alb/JCPyV T antigen primary HCC cells (Fig. 6G).

### T antigen upregulated ING5 expression by inhibiting ubiquitin-mediated degradation and promoting the T antigen-SRF-YY1-ING5 complex-associated transcription

**Figure 7:**
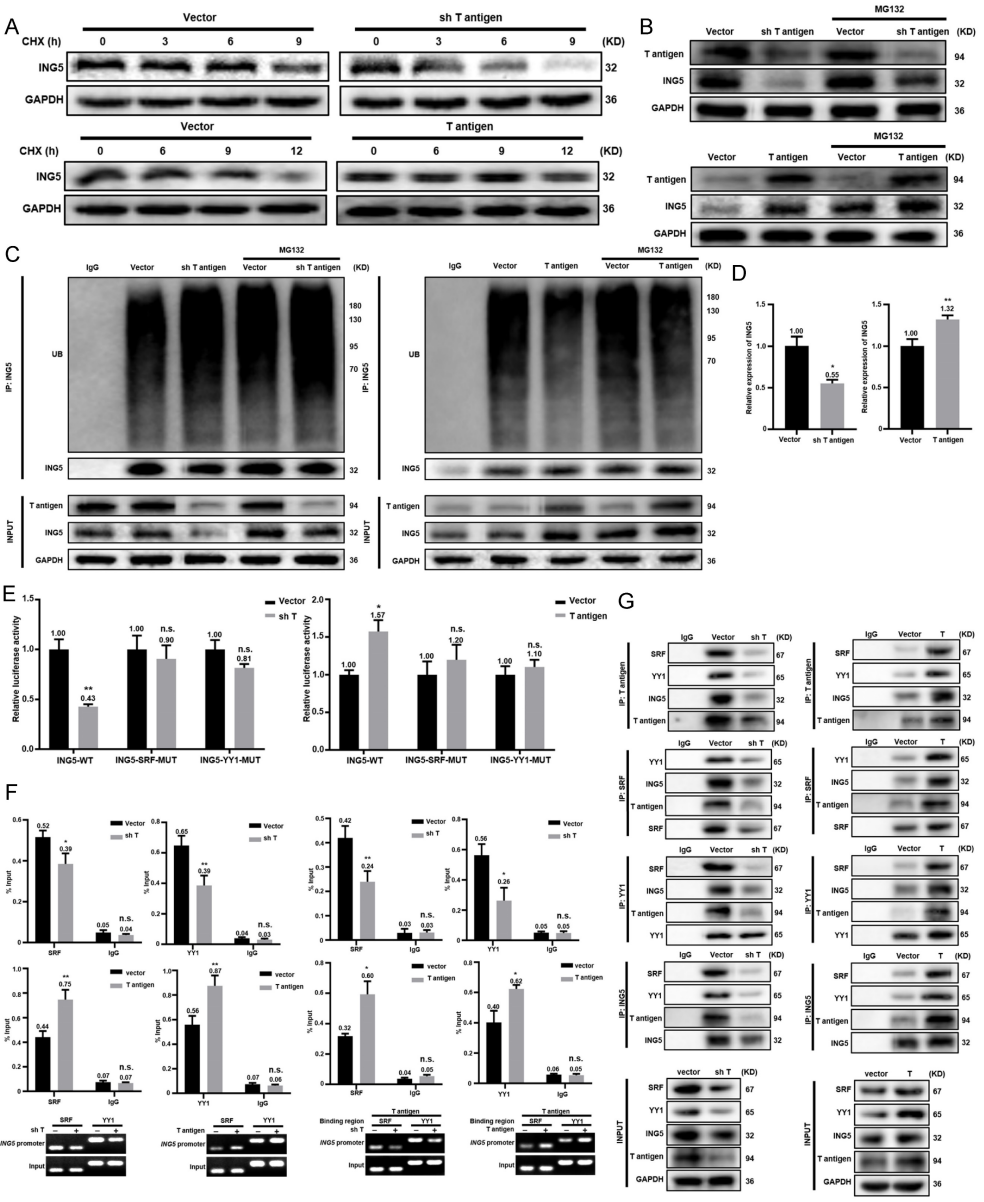
** T antigen upregulated ING5 expression by inhibiting ubiquitin-mediated degradation and promoting the T antigen-SRF-YY1-ING5 complex-associated transcription.** (A) Mouse primary HCC cells (upper) and Hepa1-6 cells (lower) were treated with CHX (0.5 μg/ml) and subjected to western blot analysis of ING5 expression. (B) Mouse primary HCC cells and Hepa1-6 cells were exposed to MG132 (10 μg/ml) for 6 h, and T antigen and ING5 expression was measured by Western blotting. (C) Cells were treated with MG132 (10 μg/ml) for 6 h, and then ING5 ubiquitination was assessed by Co-IP assays. (D) qRT‒PCR assays were performed to assess ING5 mRNA expression after silencing or overexpressing T antigen. (E) Cells were individually transfected with the pGL3-ING5 containing WT or MUT SRF/YY1 binding site for 36 h, and subjected to a dual-luciferase reporter assay. (F) ChIP assays with anti-SRF, anti-YY1 or anti-T antigen antibodies were used to measure their binding to the promoter of ING5. (G) Co-IP assays with anti-T antigen, anti-SRF, anti-YY1, and anti-ING5 antibodies were conducted to observe their interactions. Data are presented mean ± SD, and Student's t test was used for significance tests. n.s.=not significant, *P < 0.01, **P < 0.001 vs. vector group. WT, wild-type. MUT, mutant. UB, ubiquitin. IP, immunoprecipitation.

According to Western blot analysis, T antigen silencing in mouse primary HCC cells from the spontaneous HCC accelerated the degradation of ING5 protein compared to the vector control, while T antigen overexpression in Hepa1-6 cells remarkably slowed the degradation (Fig. 7A). The effect of T antigen on ING5 could be weakened by the proteasome inhibitor MG132 (Fig. 7B).

As shown in Fig. 7C, T antigen silencing enhanced the ubiquitination of ING5, which was blocked by MG132, while it was opposite for its overexpression. qRT‒PCR revealed that T antigen overexpression favored ING5 mRNA production, whereas it was converse for its knockdown (Fig. 7D). As shown in Fig. 7E, ING5 knockdown or overexpression markedly decreased or increased the promoter activity of ING5, respectively. ChIP assays demonstrated that silencing or overexpression of T antigen remarkably weakened or enhanced the binding of SRF, YY1 and T antigen to the promoter of ING5 (Fig. 7F and S3E). Co-IP assays revealed that T antigen could form a complex with SRF, YY1 and ING5 (Fig. 7G).

### UA induced HCC regression through inhibiting ING5-mediated PI3K/Akt signaling pathway *in vivo*

**Figure 8:**
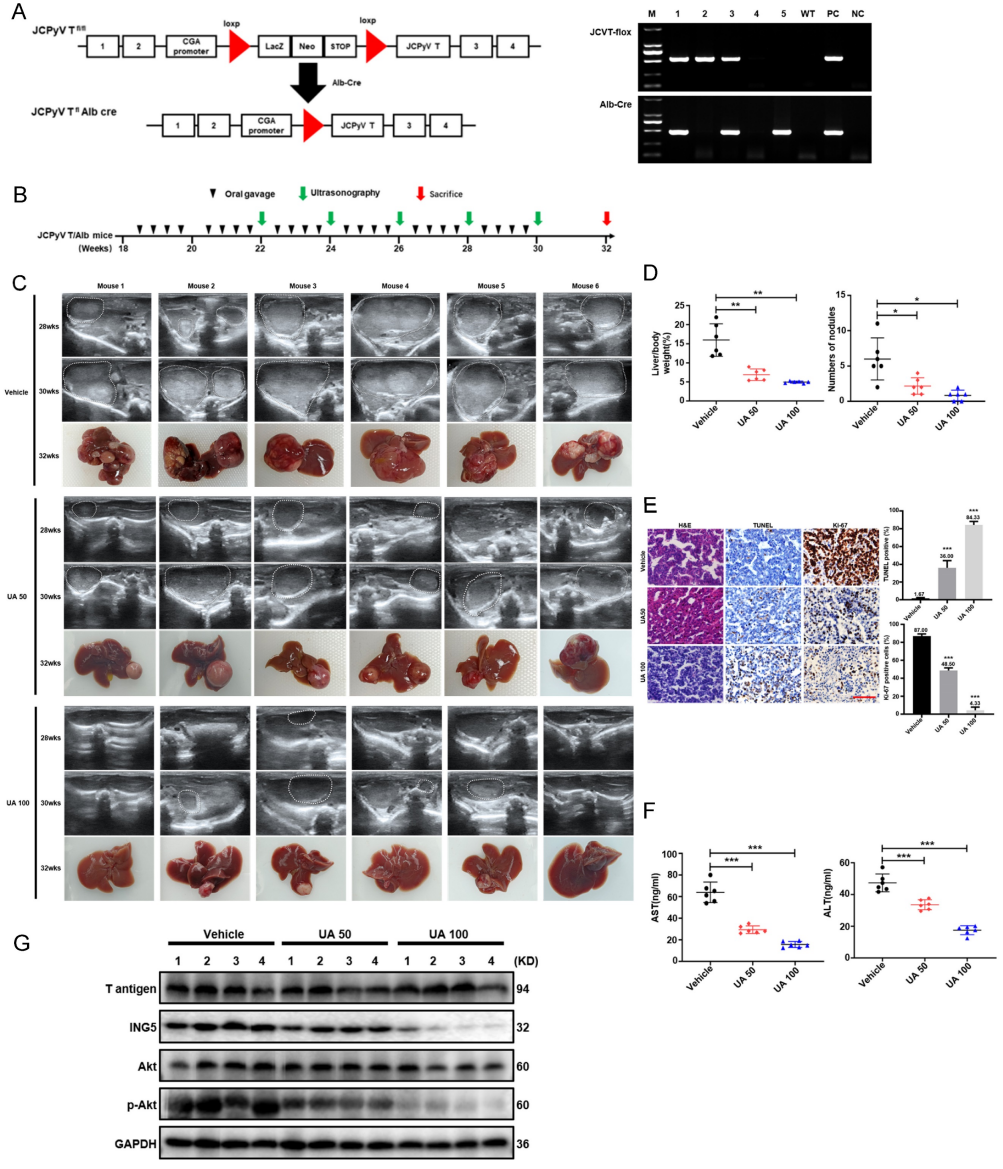
** UA suppressed the growth of spontaneous hepatocellular carcinoma by inhibiting the ING5- induced PI3K/Akt pathway.** (A) The schematic figure shows the activation of T-antigen targeted in hepatocytes with a LacZ deletion by a cre-loxp system (left) and PCR of tail DNA from Alb/JCPyV T antigen transgenic mice shows their genotype (right). (B) The schedule demonstrates that spontaneous hepatocellular carcinoma (HCC) mice were administered vehicle, 50 mg/kg, or 100 mg/kg UA at 18 weeks of age. (C) Representative ultrasonography images of mice with spontaneous HCC at 28 and 30 weeks of age, and liver images at 32 weeks of age. (D) Liver/body weight ratios and numbers of tumor nodules were measured (*P < 0.01, **P < 0.001). (E) The histological appearance, apoptosis and proliferation of HCC tissues were assessed by HE staining, TUNEL and Ki-67 immunostaining, respectively (***P < 0.001 vs. control group. Scale bar =50 μm). (F) Plasma AST and ALT levels were detected by ELISA (***P < 0.001). (G) The expression levels of T antigen, ING5, Akt and p-Akt in HCC tissues from Alb/JCPyV T antigen transgenic mice were analyzed by western blotting. Each group contained 6 mice. Differences were analyzed by Student's t test. M, marker. WT, wild-type. PC, positive control. NC, negative control. wks, weeks. TUNEL, terminal deoxynucleotidyl transferase dUTP nick end labeling.

According to the schematic diagram of T antigen activation in hepatocytes, we verified the genotype of Alb/JCPyV T antigen transgenic mice using tail DNA PCR (Fig. 8A). The transgenic mice were treated with vehicle, 50 mg/kg, or 100 mg/kg UA twice weekly by oral gavage beginning at 18 weeks of age (Fig. 8B). According to ultrasonography, HCC was detectable and grew rapidly in each of the vehicle-treated group at roughly 26 weeks of age, whereas all mice treated with 50 mg/kg UA developed HCC at about 30 weeks of age. In the high-dose (100 mg/kg) UA group, 2 of the 6 mice did not developed HCC until they were sacrificed at 32 weeks of age, and only small tumors were detected in the other mice (Fig. 8C and S4). In addition, there were significant decreases of the liver-to-body weight ratio and the number of tumor nodules in both the low- and high- dose UA groups (Fig. 8D). Histological results showed that UA treatment effectively attenuated tumor cell proliferation and promoted apoptosis (Fig. 8E). Importantly, UA effectively protected Alb/JCPyV T antigen mice from HCC-related liver injury, as evidenced by the ALT and AST levels (Fig. 8F). Western blot analysis demonstrated that UA administration significantly downregulated the levels of ING5 and its downstream p-Akt of mouse HCC tissues in a dose-dependent manner (Fig. 8G).

## Discussion

In the present study, UA not only suppressed the proliferation, migration, and invasion of HCC cells, but also induced cell apoptosis, in line with the *in vivo* results in spontaneous HCC of transgenic mice, treated with UA, suggesting the anti-tumor effects of UA. Interestingly, our results suggested that the cytotoxicity of UA was selective to HCC cells, but not to normal hepatocytes THLE-2 cells. Meanwhile, UA prominently improved liver function with the decreased serum ALT and AST levels in a spontaneous HCC mouse model, consistent with recent studies showing that UA remarkably alleviated liver injury via the modulation of gut-liver axis homeostasis and the inhibition of caspase-3 20, 21. We also showed that UA treatment clearly restrained the migration and invasion of HCC cells, as indicated by an increase in E-cadherin and a decrease in N-cadherin, demonstrating that mesenchymal-epithelial transition accounted for the inhibitory effects of UA. Additionally, a sharp reduction in the MMP was found as the number of apoptotic cells increased in response to UA. The decrease in the Bcl-2/Bax ratio also supported that UA facilitated apoptosis by the mitochondrial pathway 22. Altogether, these results may render UA an ideal candidate for HCC treatment because of its high efficiency and low toxicity.

ING5 has long been recognized as a tumor suppressor, albeit chemoresistant induction was also observed 23, 24. Previous studies showed that cytoplasmic ING5 positively correlated but nuclear ING5 negatively correlated with the tumor aggressiveness and a worse prognosis 25, 26. Qi *et al.* 27 showed that nuclear ING5 could inhibit proliferation and induce apoptosis, whereas its truncated fragments in the cytoplasm promoted senescence in tongue squamous cell carcinoma cells. In contrast, ING5 promoted the stemness and self-renewal of glioblastoma stem cells for tumor resistance and recurrence in glioblastoma by the activation of transcription of calcium channels and the follicle stimulating hormone pathway 16, in line with another report about epidermal stem cells 28. ING5 conventionally interacted with p53 as tumor suppressor via associated HAT complexes 14, 29, whereas bound to HBO128 and CDK2 in a p53-independent way to promote proliferation 13, 30. Herein, we found a higher ING5 expression in HCC than normal tissues in both mRNA and protein levels, and a positive correlation of ING5 expression with multiple malignant clinicopathological parameters including a higher serum AFP level, vascular invasion, and histological grade of tumor, as well as poor OS, PFS, RFS, and DSS of HCC patients. ING5 was found to promote cell proliferation, migration, and invasion, epithelial-mesenchymal transition, and reduced apoptosis in HCC cells, suggesting that ING5 was an oncogene and a promising therapeutic target for HCC. However, Cao *et al.* [31]and Xie *et al.* 32 reported that ING5 level was lower in HCC tissues than in normal tissues, and ING5 repressed proliferation and promoted apoptosis of HCC cells. This is contrary to our results, which may be ascribed to the differences in tissue samples and cell lines.

Mounting experimental studies have been well-documented the mechanisms of antitumor action of UA. UA could modulate Argonaute-2 to inhibit the stemness and progression of breast cancer cells 6. Moreover, UA suppressed colorectal cancer by downregulating Wnt/β-Catenin signaling pathway 7. Kim *et al.* 9 demonstrated that UA could potentially prevent the tumorigenesis of skin cancer by inhibiting DNA methyltransferases-mediated epigenetic modifications.

Regarding HCC, the antitumor mechanism of UA has been reported to be implicated in cholesterol biosynthesis, STAT3 signaling pathway, AMPKα-mediated DNA methyltransferase 1, and so on 8-11. From our data, UA was demonstrated to downregulate ING5 expression in a concentration- and time-dependent manner, with the same antitumor effects with ING5 knockdown, and ING5 silencing could weaken the effects of UA, suggesting that UA may serve as a targeted inhibitor of ING5 for HCC treatment. Aberrant activation of PI3K/Akt signaling pathway has been reported to be involved in the tumorigenesis and progression of various malignant cancers, such as HCC 33, 34. Here, ING5 was found to be positively correlated with PI3K/Akt pathway in HCC, and ING5 overexpression promoted the binding of ING5 to PI3K/Akt to increase the phosphorylation of PI3K and Akt. Interestingly, treatment with LY294002 or UA could abrogate the changes of PI3K/Akt pathway and the cell viability, apoptosis, migration as well as invasion resulting from ING5 overexpression. Taken together, UA might inhibit ING5-mediated the activation of PI3K/Akt signaling pathway to exert antitumor effects on HCC.

Our group previously reported that SRF and YY1 could bind to the promoter of ING5 gene and thereby regulated its transcription 18. In this study, the significant positive correlations among SRF, YY1 and ING5 were found, and the expression of these genes was higher in HCC than normal tissue, and positively linked to a poor prognosis of HCC patients. SRF expression has been proved to drive the hepatocarcinogenesis 35, tumor aggressiveness 36, and sorafenib resistance 37 and YY1 induced cell proliferation 38 and tumor angiogenesis 39 in HCC. Our data reported that SRF and YY1 could mediate the regulation of proliferation of HCC cells via ING5. More importantly, we also supposed that UA reduced SRF coupled with YY1 expression, and attenuated SRF-YY1 complex formation, downregulating ING5 transcription and subsequent expression.

Reportedly, gemcitabine-resistant pancreatic cancer 40, cisplatin-resistant ovarian cancer 41 and non-small cell lung cancer 42 were closely linked to *de novo* lipid synthesis. In addition, several crucial lipogenic enzymes including acetyl-CoA synthetase 2 (ACSS2) and ACC were overexpressed in cisplatin-resistant bladder cancer cells and treatment with ACSS2 inhibitor or ACSS2 siRNA could abate the cisplatin resistance 43. We also found that ING5 overexpression upregulated the expression of ACC1 and ACLY and then induced 5‑FU resistance of colorectal cells (unpublished). Here, HepG2-SR cells showed a higher expression of ING5 and lipogenic enzymes (ACC1, ACLY and MOGAT2) than the parental cells. Meanwhile, sorafenib resistance of HepG2-SR cells were closely linked to LDs formation and reversed by the knockdown of ING5 or lipogenic enzymes (ACC1 and ACLY) inhibitor, indicating that ING5 may promote lipogenesis and subsequently facilitate sorafenib resistance of HCC cells. In line with the observation of ING5 knockdown, UA was able to reverse sorafenib resistance in HCC cells by inhibiting ING5-ACC1/ACLY-LDs axis, suggesting that the combined with UA may effectively circumvent resistance to sorafenib in HCC therapy.

Our previous data reported that oncogenic T antigen protein could induce hepatocellular carcinogenesis *in vivo* 17. In the present study, we demonstrated that T antigen induced ING5 overexpression at mRNA and protein levels in HCC tissues and cells, and both ING5 and T antigen had the same oncogenic roles in HCC. T antigen downregulated BAG3 expression by inhibiting the binding of the AP2 transcription factor to BAG3 promoter 44, activated the survivin promoter and then enhanced its transcription, upregulating the expression of survivin protein 45, and dissociated β-catenin from ubiquitin-dependent proteasomal degradation by recruiting and activating Rac1, which upregulated the level of β-catenin 46. Here, we found that T antigen enhanced the levels of ING5 mRNA and protein in HCC cells. Further studies showed that T antigen interacted with ING5 promoter and promoted its transcription by forming a T antigen-SRF-YY1-ING5 complex, and inhibited the ubiquitin-mediated proteasomal degradation of ING5. Therefore, spontaneous HCC in Alb/JCPyV transgenic mice might be used to observe the antitumor effects of UA on ING5-overexpressing HCC. As expected, we found that UA could remarkably shrink the tumor volume and reduced spontaneous hepatocellular carcinogenesis via inhibiting PI3K/Akt signaling, and then be employed as a novel strategy for HCC treatment.

UA, extracted from traditional Chinese herbs, has gained much attention in recent years with its properties of multiple targets and favorable safety for HCC treatment. That said, very few preclinical or clinical studies have been conducted considering of its poor bioavailability resulting from the low water solubility 47. Therefore, synthetic analogues, nanoformulations and combination therapies of UA should be required to address this issue, which will accelerate its clinical practice for HCC patients 48-50.

**Figure 9:**
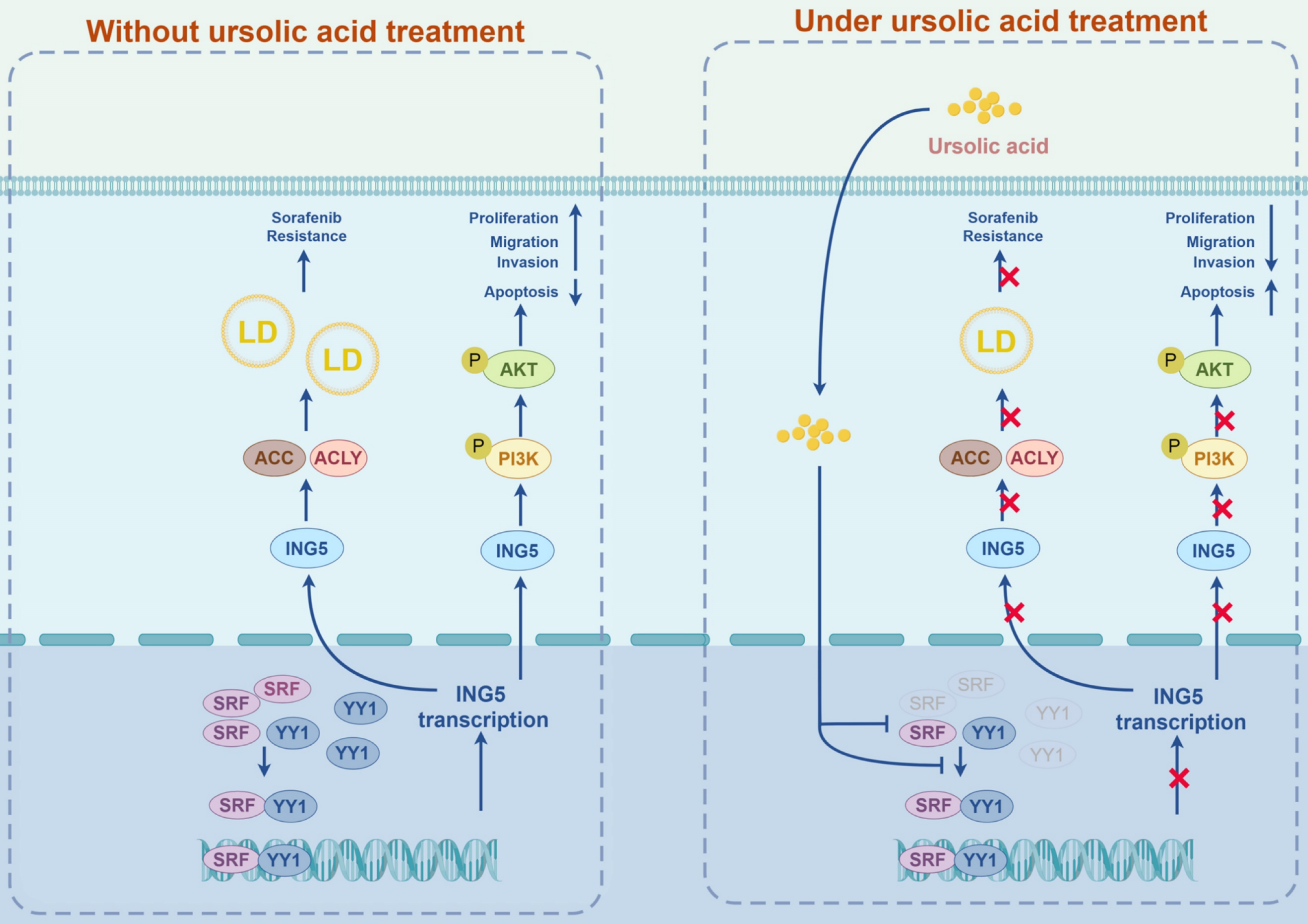
A schematic diagram of the antitumor effects of UA on HCC via targeting ING5 was generated by FigDraw (ID: ROITI9c8c8) (www.figdraw.com). LD, lipid droplet.

In conclusion, we systematically explored the antitumor effects of UA on HCC focusing on ING5 *in vitro* and *in vivo*. UA-mediated of ING5 hypoexpression was involved to inhibit carcinogenesis and progression of HCC by the inactivation of PI3K/Akt pathway. UA also reduced lipogenesis to reverse the resistance of HCC cells to sorafenib by inhibiting ING5 expression. In addition, we identified that UA downregulated SRF and YY1 expression and disassociated SRF-YY1 complex to induce ING5 hypoexpression (Fig. 9). As such, we proved that UA, a natural inhibitor of ING5, has the dual antitumoral functions of inhibiting hepatocellular carcinogenesis and reversing sorafenib resistance of HCC cells, representing a potential therapeutic strategy for HCC treatment.

## Supplementary Material

Supplementary figures and tables.

## Abbreviations

ING5: inhibitor of growth 5
UA: ursolic acid
HCC: hepatocellular carcinoma
HAT: histone acetyltransferase
HDAC: histone deacetylase
PHD: plant homeodomain
SRF: serum response factor
YY1: Yin Yang-1
FBS: fetal bovine serum
ACLY: ATP-citrate lyase
ACC1: acetyl-CoA carboxylase 1
CHX: Cycloheximide
qRT-PCR: quantitative real-time PCR
CCK-8: cell counting kit-8
MMP: mitochondrial membrane potential
Co-IP: Co-immunoprecipitation
ChIP: chromatin immunoprecipitation
WT: wild-type
MUT: mutant
HE: hematoxylin and eosin
IHC: immunohistochemistry
TUNEL: terminal deoxynucleotidyl transferase dUTP nick end labeling
ELISA: Enzyme-linked immunosorbent assay
ALT: alanine aminotransferase
AST: aspartate aminotransferase
HPA: Human Protein Atlas
GEO: Gene Expression Omnibus
KM: Kaplan-Meier
ANOVA: one-way analysis of variance
SD: standard deviation
AFP: alpha-fetoprotein
OS: overall survival
PFS: progression-free survival
RFS: relapse-free survival
DSS: disease-specific survival
LDs: lipid droplets
HG: high glucose
PA: palmitic acid
ACSS2: acetyl-CoA synthetase 2
IP: immunoprecipitation
HR: hazard ratio
Sor: sorafenib
UB: ubiquitin
PC: positive control
NC: negative control
