# Supplementary material for: The Antitumor and Sorafenib-resistant Reversal Effects of Ursolic Acid on Hepatocellular Carcinoma via Targeting ING5
Source: Int J Biol Sci. 2024 Aug 5;20(11):4190–208. doi: 10.7150/ijbs.97720 (PMC11379078; doi:10.7150/ijbs.97720)
Supplement: Supplementary file 1 — Supplementary figures and tables. [file ijbsv20p4190s1.pdf]

**Supplementary figure legends**

**Figure S1.**

(A) The expression of ING5 protein in hepatocellular carcinoma (HCC) tissue (patient ID: 2766) and normal liver tissue (patient ID: 3402) was obtained from the HPA database (https://www.proteinatlas.org/). (B) The mRNA and protein expression of ING5 in normal and HCC patients was assessed by TCGA-LIHC and TCGA-CPTAC data obtained from the UALCAN and GEO (GSE102079 and GSE76427) databases. (C) KM analyses of OS, PFS, RFS and DSS of HCC patients based on ING5 expression were performed using KM Plotter (http://kmplot.com/). ING5 expression in HepG2 and PLC/PRF/5 cells was validated by western blot (D) and qRT–PCR (E) (n=3). All data are presented as mean ± SD, Student's t test was used for significance tests, *P < 0.05, ***P < 0.001 vs. the vector group. Scale bar=30 μm. HR, hazard ratio. OS, overall survival. PFS, progression-free survival. RFS, relapse-free survival. DSS, disease-specific survival.

**Figure S2.**

Cell viability (A), apoptosis (B) (n=3), migration (C) (n=5), and invasion (D) (n=3) were analyzed in HepG2 and PLC/PRF/5 cells after ING5 silencing or/and treatment with 5 μM UA (*P < 0.05, **P < 0.01, ***P < 0.001. Scale bar =50 μm).

**Figure S3.**

(A) HepG2-SR and parental cells were treated with sorafenib for 24 h, after which cell viability was analyzed by CCK-8 assays. (B) HepG2-SR cells were treated with UA for 24 h, after which cell viability was analyzed by CCK-8 assays. (C) The transfected cells were validated using western blotting. (D) HepG2 cells were exposed to culture medium containing 2% and 12% FBS for 36 h, respectively. HepG2 cells were transfected with ING5 shRNA, SRF or YY1 siRNA following exposure to medium containing 12% FBS for 36 h. HepG2 cells were transfected with ING5 overexpression plasmid following exposure to medium containing 2% FBS for 36 h. Colony formation assays were performed for assessing the clonogenicity of HepG2 cells (n=3, *P < 0.05, **P < 0.01). (E) ChIP assays with anti-IgG and anti-histone H3 antibodies were used as negative and positive controls, respectively.

**Figure S4.**

Ultrasonography was performed to visualize the livers of spontaneous HCC in mice at 22, 24 and 26 weeks of age.

# Figure S1

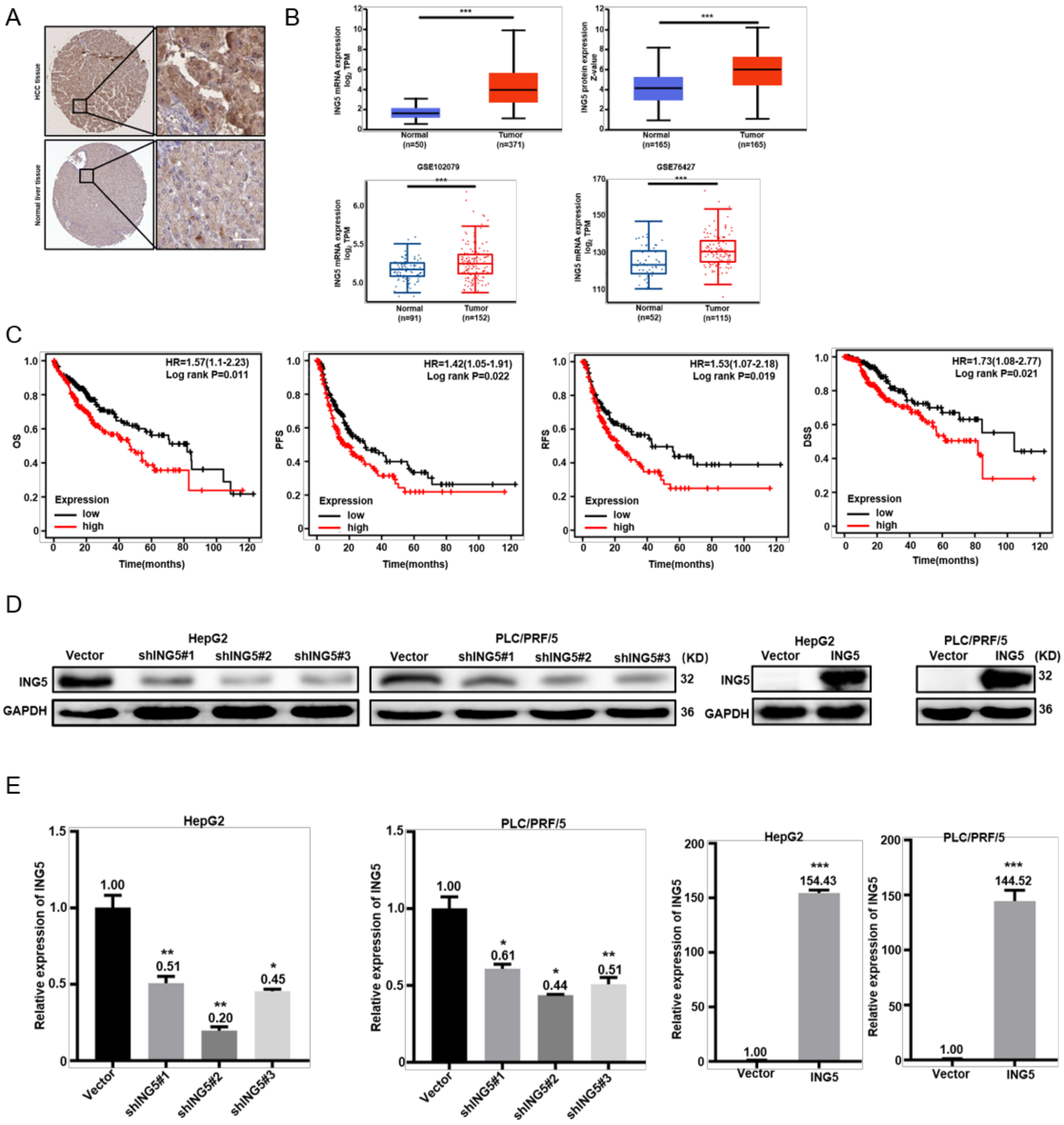
A
HCC tissue
Normal liver tissue
B
TCGA-LIHC
TCGA-CPTAC
GSE102079
GSE76427
Normal
Tumor
C
Expression
low
high
Time(months)
HR=1.57(1.1-2.23)
Log rank P=0.011
HR=1.42(1.05-1.91)
Log rank P=0.022
HR=1.53(1.07-2.18)
Log rank P=0.019
HR=1.73(1.08-2.77)
Log rank P=0.021
D
HepG2
PLC/PRF/5
Vector
shING5#1
shING5#2
shING5#3
ING5
GAPDH
(KD)
E
Relative expression of ING5

# Figure S2

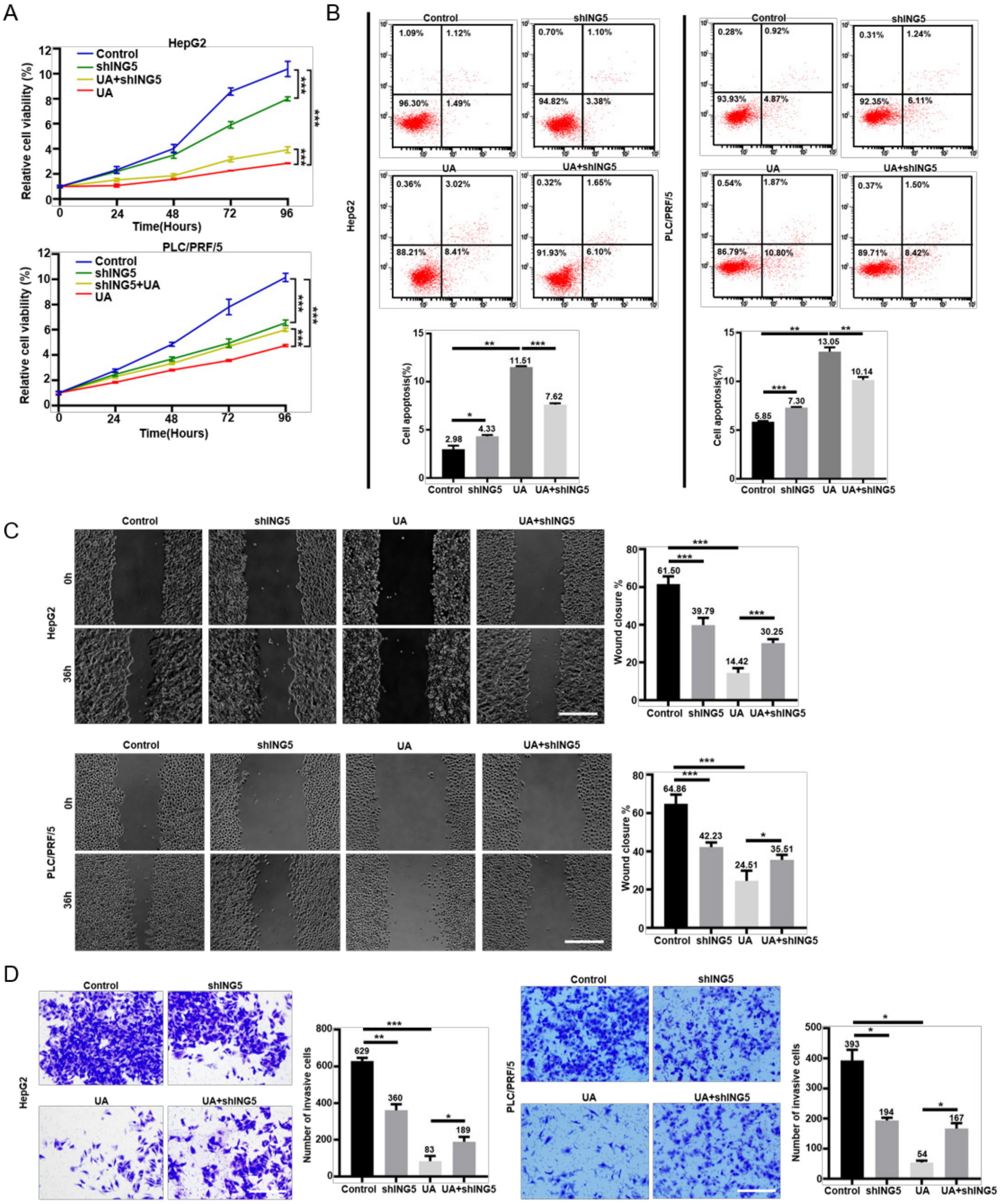
A
HepG2
Control
shING5
UA+shING5
UA
Relative cell viability (%)
Time(Hours)
PLC/PRF/5
shING5+UA
B
Control
shING5
UA
UA+shING5
HepG2
PLC/PRF/5
Cell apoptosis(%)
C
0h
36h
Wound closure %
D
Number of invasive cells

# Figure S3

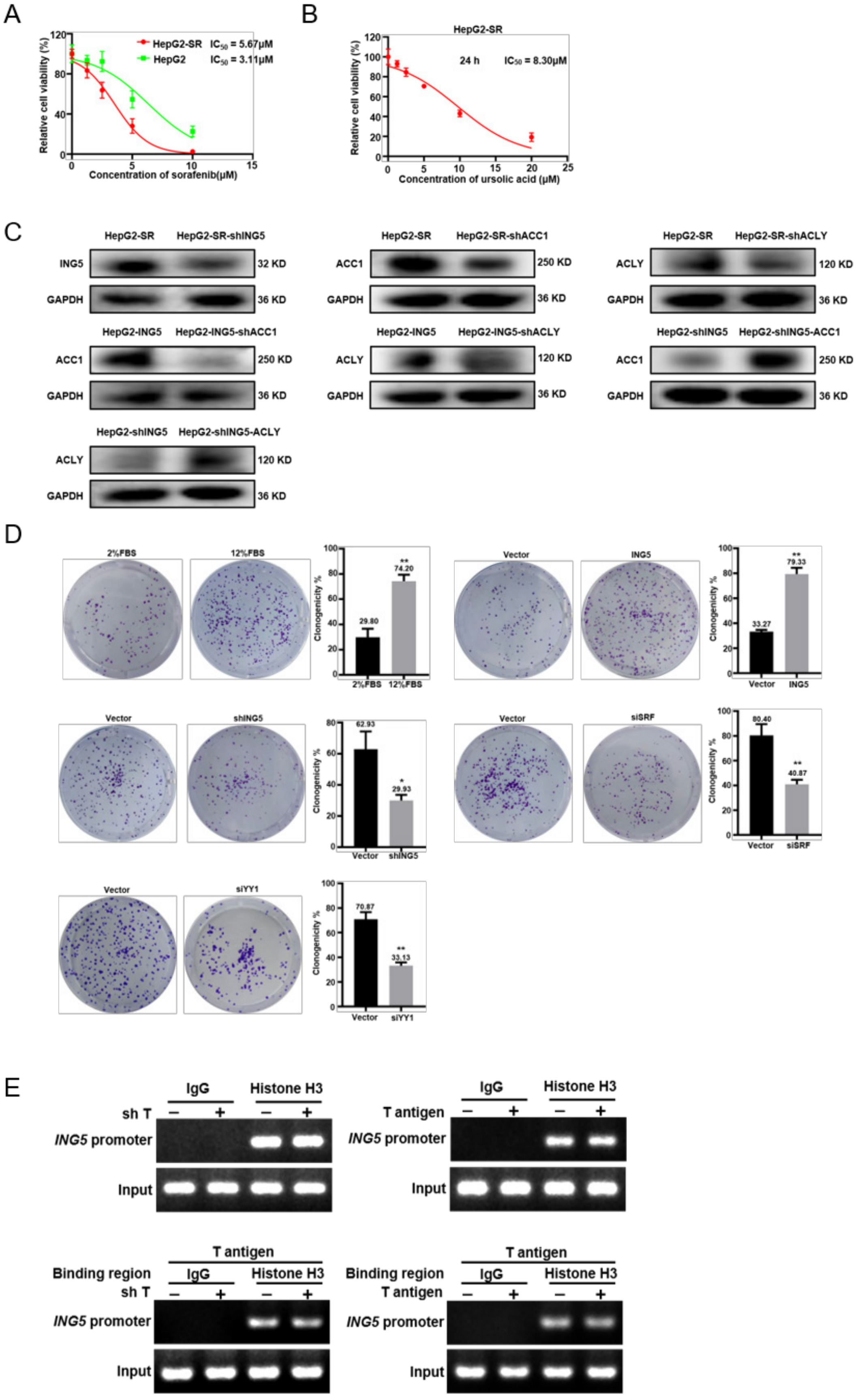
A
HepG2-SR IC50 = 5.67μM
HepG2 IC50 = 3.11μM
Relative cell viability (%)
Concentration of sorafenib(μM)
B
HepG2-SR
24 h IC50 = 8.30μM
Relative cell viability (%)
Concentration of ursolic acid (μM)
C
HepG2-SR HepG2-SR-shING5
ING5 32 KD
GAPDH 36 KD
HepG2-SR HepG2-SR-shACC1
ACC1 250 KD
GAPDH 36 KD
HepG2-SR HepG2-SR-shACLY
ACLY 120 KD
GAPDH 36 KD
HepG2-ING5 HepG2-ING5-shACC1
ACC1 250 KD
GAPDH 36 KD
HepG2-ING5 HepG2-ING5-shACLY
ACLY 120 KD
GAPDH 36 KD
HepG2-shING5 HepG2-shING5-ACC1
ACC1 250 KD
GAPDH 36 KD
HepG2-shING5 HepG2-shING5-ACLY
ACLY 120 KD
GAPDH 36 KD
D
2%FBS 12%FBS
Clonogenicity %
29.80 74.20
Vector ING5
33.27 79.33
Vector shING5
62.93 29.93
Vector siSRF
80.40 40.87
Vector siYY1
70.87 33.13
E
IgG Histone H3
sh T
ING5 promoter
Input
IgG Histone H3
T antigen
ING5 promoter
Input
T antigen
Binding region
IgG Histone H3
sh T
ING5 promoter
Input
T antigen
Binding region
IgG Histone H3
T antigen
ING5 promoter
Input

## Figure S4

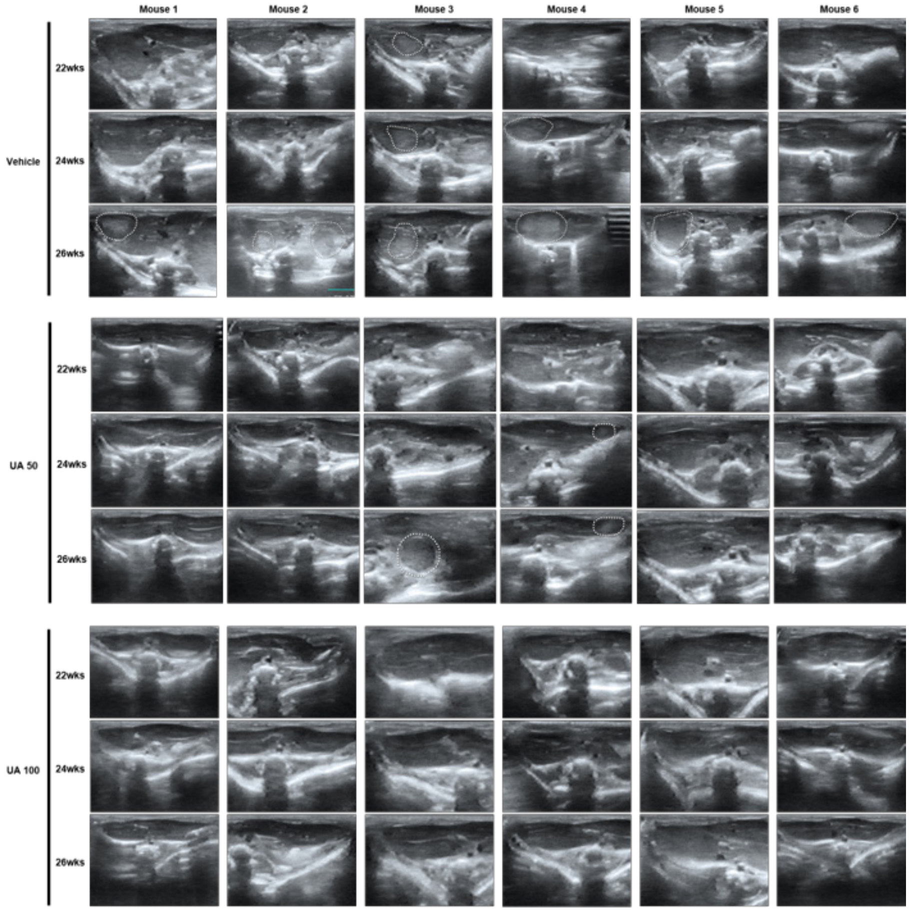
Mouse 1
Mouse 2
Mouse 3
Mouse 4
Mouse 5
Mouse 6
Vehicle
22wks
24wks
26wks
UA 50
22wks
24wks
26wks
UA 100
22wks
24wks
26wks

## Supplementary tables

**Table S1. Primary antibodies used in this study.**

| Antibody | Manufacturer | Catalog Number | Application |
|---|---|---|---|
| PCNA | Proteintech | 60097-1-Ig | 1:5000 for WB |
| Bax | Proteintech | 50599-2-Ig | 1:2000 for WB |
| Bcl-2 | Proteintech | 26593-1-AP | 1:1000 for WB |
| E-cadherin | Bioss | bs-1519R | 1:500 for WB |
| N-cadherin | Bioss | bs-1172R | 1:500 for WB |
| MMP-9 | Santa Cruz | sc-21733 | 1:100 for WB |
| Cyclin B1 | Santa Cruz | sc-245 | 1:100 for WB |
| GAPDH | Proteintech | 60004-1-Ig | 1:20000 for WB |
| ING5 | Proteintech | 10665-1-AP | 1:500 for WB |
| | | | 1: 20 for Co-IP |
| PI3K | Proteintech | 60225-1-Ig | 1:500 for WB |
| | | | 1: 20 for Co-IP |
| p-PI3K | Cell Signaling Technology | 17366S | 1:1000 for WB |
| Akt | Proteintech | 10176-2-AP | 1:2000 for WB |
| | | | 1: 20 for Co-IP |
| p-Akt | Proteintech | 28731-1-AP | 1:1000 for WB |
| SRF | Cell Signaling Technology | 5147S | 1:1000 for WB |
| | | | 1:50 for Co-IP |
| | | | 1:100 for ChIP |
| YY1 | Cell Signaling Technology | 63227S | 1:1000 for WB |
| | | | 1:50 for Co-IP |
| | | | 1:100 for ChIP |

| | | | |
|---|---|---|---|
| ACC1 | Proteintech | 21923-1-AP | 1:1000 for WB |
| ACLY | Proteintech | 15421-1-AP | 1:1000 for WB |
| MOGAT2 | Proteintech | 21286-1-AP | 1:500 for WB |
| SV40 T Antigen | Merck Calbiochem | PAb416 | 1:100 for WB |
| | | | 1:20 for Co-IP |
| | | | 1:50 for ChIP |
| Ubiquitin | Proteintech | 10201-2-AP | 1:1000 for WB |
| Ki-67 | Cell Signaling Technology | 12202S | 1:300 for IHC |

**Table S2. Primer sequences used in this study.**

| Target | | Sequence (5'-3') |
|---|---|---|
| Human ING5 | Forward | GAGGACATCAGAGGAAGACACAC |
| | Reverse | CACTCAATTGGACAGTCTGGATT |
| Mouse ING5 | Forward | AGACTTGATGCTGACCTGGC |
| | Reverse | TTTTCAAGCTCCGTGCTCCA |
| Human SRF | Forward | CACAACAGACCAGAGAATGAGTG |
| | Reverse | GTAGAGGTGCTAGGTGCTGTTTG |
| Mouse SRF | Forward | CGTCACCCTGGACAGACG |
| | Reverse | TCCTTCGGCCAGTCAGTGT |
| Human YY1 | Forward | ACGGCTTCGAGGATCAGATTC |
| | Reverse | TGACCAGCGTTTGTTCAATGT |
| Mouse YY1 | Forward | GCCCCTTCGACGGTTGTAAT |
| | Reverse | CACTCCTGAAGAGGCTTCCC |
| Human GAPDH | Forward | CAATGACCCCTTCATTGACC |
| | Reverse | TGGAAGATGGTGATGGGATT |
| Mouse GAPDH | Forward | GGGTCCCAGCTTAGGTTCAT |
| | Reverse | TACGGCCAAATCCGTTCACA |

**Table S3. Tumor incidence in Alb/JCPyV T antigen transgenic mice across different weeks of age.**

| Sex | Age (in weeks) | Total incidence |
|---|---|---|
| **Male** | 24 | 100% (8/8) |
| | 20 | 87.5% (7/8) |
| | 16 | 50% (4/8) |
| | 12 | 12.5% (1/8) |
| **Female** | 32 | 100% (8/8) |
| | 28 | 62.5% (5/8) |
| | 24 | 50% (4/8) |
| | 20 | 25% (2/8) |
| | 16 | 0 (0/8) |

**Table S4. The clinicopathological significance of ING5 mRNA expression in hepatocellular carcinoma patients.**

| Characteristic | Low expression | High expression | P-value |
|---|---|---|---|
| **Age, n (%)** | | | 0.088 |
| <=60 | 80 (21.4%) | 97 (26%) | |
| >60 | 107 (28.7%) | 89 (23.9%) | |
| **Gender, n (%)** | | | 0.658 |
| Female | 58 (15.5%) | 63 (16.8%) | |
| Male | 129 (34.5%) | 124 (33.2%) | |
| **Race, n (%)** | | | **0.048** |
| Asian | 67 (18.5%) | 93 (25.7%) | |
| Black or African American | 8 (2.2%) | 9 (2.5%) | |
| White | 102 (28.2%) | 83 (22.9%) | |
| **Weight, n (%)** | | | **< 0.001** |
| <=70 | 76 (22%) | 108 (31.2%) | |
| >70 | 97 (28%) | 65 (18.8%) | |
| **T stage, n (%)** | | | 0.456 |
| T1 | 97 (26.1%) | 86 (23.2%) | |
| T2 | 46 (12.4%) | 49 (13.2%) | |
| T3 | 34 (9.2%) | 46 (12.4%) | |
| T4 | 7 (1.9%) | 6 (1.6%) | |
| **N stage, n (%)** | | | 1.000 |
| N0 | 117 (45.3%) | 137 (53.1%) | |
| N1 | 2 (0.8%) | 2 (0.8%) | |

| | | | |
|---|---|---|---|
| **M stage, n (%)** | | | 0.358 |
| M0 | 129 (47.4%) | 139 (51.1%) | |
| M1 | 3 (1.1%) | 1 (0.4%) | |
| **Pathologic stage, n (%)** | | | 0.293 |
| Stage I | 91 (26%) | 82 (23.4%) | |
| Stage II | 42 (12%) | 45 (12.9%) | |
| Stage III | 37 (10.6%) | 48 (13.7%) | |
| Stage IV | 4 (1.1%) | 1 (0.3%) | |
| **Tumor status, n (%)** | | | **0.005** |
| Tumor free | 115 (32.4%) | 87 (24.5%) | |
| With tumor | 63 (17.7%) | 90 (25.4%) | |
| **Residual tumor, n (%)** | | | 0.714 |
| R0 | 162 (47%) | 165 (47.8%) | |
| R1 | 9 (2.6%) | 8 (2.3%) | |
| R2 | 1 (0.3%) | 0 (0%) | |
| **Histologic grade, n (%)** | | | **< 0.001** |
| G1 | 32 (8.7%) | 23 (6.2%) | |
| G2 | 103 (27.9%) | 75 (20.3%) | |
| G3 | 46 (12.5%) | 78 (21.1%) | |
| G4 | 3 (0.8%) | 9 (2.4%) | |
| **AFP (ng/ml), n (%)** | | | **< 0.001** |
| <=400 | 132 (47.1%) | 83 (29.6%) | |
| >400 | 14 (5%) | 51 (18.2%) | |
| **Child-Pugh grade, n (%)** | | | 0.811 |
| A | 119 (49.4%) | 100 (41.5%) | |
| B | 11 (4.6%) | 10 (4.1%) | |

| | | | |
|---|---|---|---|
| C | 0 (0%) | 1 (0.4%) | |
| **Vascular invasion, n (%)** | | | **0.035** |
| No | 118 (37.1%) | 90 (28.3%) | |
| Yes | 48 (15.1%) | 62 (19.5%) | |

AFP, alpha-fetoprotein.

**Table S5. The prognostic significance of ING5 mRNA expression in hepatocellular carcinoma patients**

| Clinicopathological features | Overall survival | | Progression-free survival | | Relapse-free survival | |
|---|---|---|---|---|---|---|
| | HR (CI 95%) | P-value | HR (CI 95%) | P-value | HR (CI 95%) | P-value |
| **Total** | 1.57 (1.1-2.23) | **0.0112** | 1.42 (1.05-1.91) | **0.0223** | 1.53 (1.07–2.18) | **0.0187** |
| **Stage** | | | | | | |
| 1 | 1.79(0.98-3.29) | 0.0554 | 1.39(0.83-2.36) | 0.212 | 1.52(0.86-2.69) | 0.1457 |
| 1-2 | 1.69(1.05-2.74) | 0.303 | 1.3(0.88-1.9) | 0.1824 | 1.47(0.94-2.29) | 0.0883 |
| 2 | 2.49(1.09-5.68) | 0.246 | 2.3(1.09-4.88) | **0.0253** | 2.36(1.01-5.55) | **0.0422** |
| 2-3 | 2.2(1.33-3.63) | **0.0017** | 2.13(1.28-3.54) | **0.0028** | 1.91(1.16-1.31) | **0.0096** |
| 3 | 2.16(1.15-4.08) | **0.0149** | 2.4(1.24-4.63) | **0.0075** | 1.89(0.94-3.81) | 0.0701 |
| 3-4 | 2.07(1.14-3.75) | **0.0141** | 2.14(1.15-3.96) | **0.0139** | 1.89(0.94-3.81) | 0.0701 |
| 4 | —— | —— | —— | —— | —— | —— |
| **Grade** | | | | | | |
| 1 | 2.54(0.96-6.75) | 0.0534 | 3.22(1.41-3.73) | **0.0036** | 3.12(1.1-8.87) | **0.0249** |
| 2 | 1.3(0.77-2.18) | 0.323 | 1.23(0.79-1.89) | 0.3579 | 1.39(0.82-2.36) | 0.2183 |
| 3 | 2.65(1.27-5.54) | **0.0072** | 1.61(0.89-2.94) | 0.1137 | 1.35(0.76-2.37) | 0.3019 |
| 4 | —— | —— | —— | —— | —— | —— |
| **AJCC_T** | | | | | | |
| 1 | 1.65(0.93-2.95) | 0.0865 | 1.34(0.8-2.23) | 0.2653 | **1.5(0.86-2.61)** | 0.1514 |
| 2 | 2.3(1.09-4.87) | **0.0247** | 2.23(1.13-4.4) | **0.0184** | 2.36(1.07-5.25) | **0.0297** |
| 3 | 2.88(1.32-6.29) | **0.0058** | 1.94(0.98-3.84) | 0.0521 | 1.56(0.77-3.17) | 0.2112 |
| 4 | —— | —— | —— | —— | —— | —— |
| **Vascular invasion** | | | | | | |

| | | | | | | |
|---|---|---|---|---|---|---|
| none | 1.51(0.86-2.64) | 0.148 | 1.21(0.77-1.89) | 0.4035 | 1.3(0.8-2.11) | 0.2933 |
| micro | 1.53(0.71-3.28) | 0.2725 | 2.09(0.93-4.67) | 0.0665 | 2.29(1.08-4.85) | **0.0263** |
| macro | —— | —— | —— | —— | —— | —— |
| **Gender** | | | | | | |
| male | 1.83(1.17-2.87) | **0.0071** | 1.7(1.18-2.46) | 0.0042 | 1.61(1.04-2.47) | **0.0294** |
| female | 1.7(0.91-3.17) | 0.0894 | 0.74(0.44-1.25) | 0.2636 | 1.47(0.79-2.72) | 0.2223 |
| **Race** | | | | | | |
| white | 1.88(1.183-2.98) | **0.0067** | 1.44(0.97-2.16) | 0.0721 | 1.7(1.05-2.77) | **0.0303** |
| black or african american | —— | —— | —— | —— | —— | —— |
| asian | 1.85(1.02-3.37) | **0.0404** | 1.71(1.02-2.85) | 0.0393 | 1.62(0.94-2.8) | 0.0793 |
| **Sorafenib treatment** | | | | | | |
| treated | 2.54(0.79-8.2) | 0.107 | 1.8(0.79-4.08) | 0.1558 | 0.69(0.28-1.67) | 0.4065 |
| **Alcohol consumption** | | | | | | |
| yes | 3.8(1.92-7.53) | **4.6e-5** | 2.18(1.16-4.08) | **0.0137** | 2.16(1.01-4.63) | **0.0422** |
| none | 1.5(0.94-2.4) | 0.0885 | 1.45(0.96-2.2) | 0.0728 | 1.62(1.02-2.56) | **0.0374** |
| **Hepatitis virus** | | | | | | |
| yes | 1.48(0.77-2.86) | 0.2383 | 1.6(1-2.57) | 0.0501 | 1.54(0.92-2.6) | 0.0997 |
| none | 2.22(1.41-3.51) | **0.0005** | 1.8(1.11-2.9) | **0.0158** | 2.26(1.26-4.07) | **0.0054** |

HR, hazard ratio. CI, confidence interval.
